# Nonmonotonic scaling of the anomalous Hall effect in a bicollinear antiferromagnet

**DOI:** 10.1126/sciadv.aef9859

**Published:** 2026-09-30

**Authors:** Ruifeng Wang, Chi Fang, Ilya Kostanovski, Ke Xiao, Felix Küster, Jenny Davern, Naoto Nagaosa, Stuart S. P. Parkin

**Affiliations:** ^1^ Max Planck Institute of Microstructure Physics, Halle (Saale) 06120, Germany.; ^2^ RIKEN Center for Emergent Matter Science (CEMS), Wako 351-0198, Japan.; ^3^ Fundamental Quantum Science Program (FQSP), TRIP Headquarters, RIKEN, Wako 351-0198, Japan.

## Abstract

An anomalous Hall effect (AHE) in antiferromagnetic (AF) systems without net magnetization is of considerable interest for fundamental physics and spintronic applications. Of particular interest is the two-dimensional van der Waals antiferromagnet iron telluride (FeTe), which has an unusual magnetically compensated bicollinear AF structure and claimed Kondo interactions that cause strong band renormalization. Here, we investigate the AHE in epitaxial FeTe thin films grown by molecular beam epitaxy. A large anomalous Hall conductivity is exhibited below the Néel temperature (*T*_N_ ∼ 60 kelvins) and, notably, becomes nonlinear at high magnetic fields within a narrow temperature window around 49 kelvins, deviating from conventional AHE scaling behavior versus the longitudinal conductivity. The accompanying field-induced canted magnetic moment activates these AHE responses by tuning the Berry curvature derived from FeTe’s topological band structure once the zero-field symmetry constraint is lifted. The unconventional scaling of AHE highlights the intricate interplay between antiferromagnetism, topology, and electronic transport in FeTe.

## INTRODUCTION

The anomalous Hall effect (AHE) has long been recognized as a fundamental transport phenomenon that reveals the intimate connection between magnetism and electronic properties in solids. In general, the AHE can originate from an extrinsic mechanism due to scattering from impurities and defects, such as skew scattering and side jump, or from an intrinsic mechanism derived from the Berry curvature of the electronic bands (*1*). In ferromagnetic (FM) systems, both mechanisms usually coexist, and, as a result, the intrinsic contribution to the AHE is often obscured and requires further distinction from extrinsic contributions (*2*, *3*). This has encouraged extensive investigations into the scaling laws between the anomalous Hall conductivity (AHC) σ*_xy_* and the longitudinal conductivity σ*_xx_* in a variety of FM materials. Typically, σ*_xy_* ∝ σ*_xx_* for skew scattering whereas σ*_xy_* remains nearly constant for the side jump and intrinsic mechanisms. Together, these yield a monotonic conductivity dependence below the magnetic ordering temperature and are well explained by quantum transport theory (*4*). Meanwhile, an AHE has also been reported in several distinct classes of antiferromagnetic (AF) systems (*5*–*13*), including noncollinear antiferromagnets (*8*–*10*), noncoplanar spin systems, and, more recently, collinear or altermagnetic materials (*12*, *13*). With vanishing net magnetization, these systems still maintain broken symmetries and may generate large Berry curvature, making it possible to realize an AHE that is dominantly intrinsic in origin. As a result, although the AHE in AF systems cannot be reliably inferred from their magnetization, the developing scaling analysis could instead provide a framework for understanding the coexistence of AHE mechanisms in AF materials (*14*–*16*).

Additionally, deviations from the aforementioned conventional scaling relation probably indicate the involvement of additional contributions (*17*–*21*). For instance, an obvious breakdown of scaling laws is reported in the FM USbTe with strong electron correlations (*18*). A Kondo lattice, where the array of local moments interacts coherently with the conduction electrons via an AF interaction (*22*), can generate flat bands and thereby effectively modify the Berry curvature as the temperature varies. The extrinsic mechanism, in this case, is also in competition, making the analysis of the scaling relationship more complicated (*18*). Recently, similar Kondo lattice behavior has been found in several AF systems, e.g., CeTe_3_ (*23*), CeIn_3_ (*24*), UOTe (*25*), Mn_3+*x*_Sn_1−*x*_ (*26*), and FeTe (*27*–*29*). But the AHE and its scaling relations in these systems have not yet been fully realised. Among them, tetragonal FeTe (Fig. 1A) represents a particularly intriguing case (*27*, *30*) with a high Kondo temperature (*27*). Upon cooling below its Néel temperature, *T*_N_, FeTe transforms from a tetragonal P4/nmm into a monoclinic P2_1_/m structure and develops a commensurate bicollinear antiferromagnetic (BCAF) order with a propagation vector **q** = (^1^/_2_, 0, ^1^/_2_) (Fig. 1B) (*31*, *32*). Because the combined P⊗T symmetry is retained, where P is the spatial-inversion symmetry and T is the time-reversal symmetry, a twofold Kramers’ degeneracy is enforced across all bands and the cancellation of Berry curvatures forbids a spontaneous intrinsic AHE (Fig. 1C). At the same time, FeTe has a topologically nontrivial band structure featuring nodal lines and nodal points near the Fermi energy (*33*, *34*), which can, in principle, allow an uncompensated Berry-curvature contribution to the Hall conductivity (HC) once the relevant symmetry constraints are lifted, for example, by magnetic field–induced spin canting (Fig. 1D) (*10*, *35*). In real samples, imperfections in the layers (*36*, *37*) or interstitial Fe atoms (*38*) may additionally generate a net magnetization and the AHE in low magnetic fields (<2 T). Nevertheless, the relationship between the BCAF order and the topological band structure governing the AHE intrinsically in FeTe has not yet been established.

**Fig. 1.**
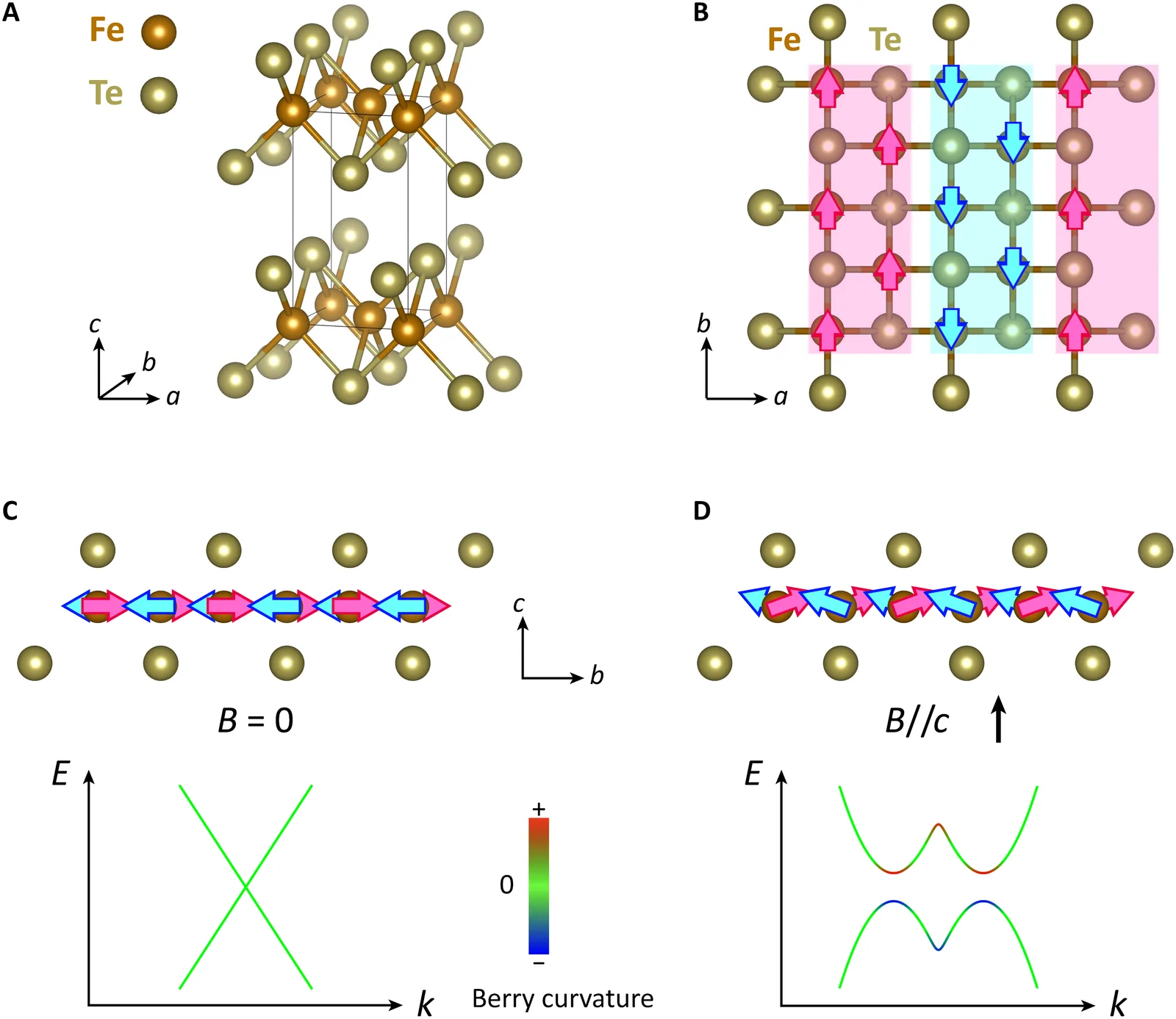
Structural models and schematic mechanism of the field-induced intrinsic Hall response in FeTe. (**A**) Schematic lattice structure of FeTe. (**B**) Schematic diagram of the bicollinear AF order in the ground state on the *ab* plane of FeTe. The Fe spins are marked by red and blue arrows. (**C**) Schematic spin configuration and band structure at *B* = 0. The collinear spin configuration preserves the protecting symmetry, resulting in symmetry-protected band crossings and zero net Berry curvature. (**D**) Corresponding schematic diagram under *B*//*c*. The out-of-plane magnetic field induces spin canting, lifts the symmetry protection, and opens gaps at the band crossings, thereby generating finite net Berry curvature. The band colors represent the sign of the Berry curvature.

Here, we report the observation of an AHE, driven by the intrinsic Berry curvature, and its nonmonotonic scaling relation in epitaxial films of BCAF FeTe in the presence of a magnetic field. High-quality single-crystal FeTe thin films were prepared by molecular beam epitaxy on (001) SrTiO_3_ single-crystalline substrates. These films exhibit a Néel temperature (*T*_N_) of ∼60 K, similar to that reported in bulk single crystals. They exhibit a BCAF order below *T*_N_, which arises from a competition between first-, second-, and third-nearest–neighbor magnetic exchange interactions among Fe atoms that form a face-centered square lattice within a single plane (*39*, *40*). These planes are separated from one another by planes of Te atoms which themselves are separated from each other by the van der Waals (vdW) gap. Above *T*_N_, tetragonal FeTe exhibits a HC that is proportional to the magnetic field with a positive slope. However, in a specific temperature range just below *T*_N_, the slope of the HC changes sign, accompanied by the emergence of a nonlinear AHC at high fields, in line with a recent theoretical calculation result (*35*). These results highlight a remarkable instance of pronounced AHE governed by intrinsic mechanisms in a collinear AF material.

## RESULTS

### Preparation of single-crystal bicollinear FeTe films

High-quality FeTe films were prepared and precisely controlled using a state-of-the-art VEECO molecular beam epitaxy system (see Materials and Methods). Under optimized growth parameters, FeTe films were epitaxially grown on SrTiO_3_ (001) (STO) substrates in the Volmer-Weber mode, as evidenced by sharp reflection high-energy electron diffraction (RHEED) patterns and clear surface atomic force microscopic images (see fig. S1). The surface topography of FeTe films (fig. S1, B to E) reveals uniformly oriented tetragonal islands coalesced into a continuous thin film that fully covers the STO substrate. Further analysis highlights well-defined terrace edges with a pronounced fourfold symmetry, indicative of the high single-crystal quality of the FeTe film in the tetragonal structure. The BCAF order exists in a narrow growth window (*41*–*44*). Rutherford backscattering spectrometry (RBS) revealed the stoichiometry of our films to be Fe_1.08_Te (fig. S2), consistent with results on bulk Fe_1+*x*_Te (*41*, *42*), which only forms and maintains BCAF ordering within the composition range 0.05 < *x* < 0.12. Unless otherwise specified, the transport and magnetic measurements were performed on 25-monolayer (ML) thick films, corresponding to an RBS-determined thickness of 15.3 ± 2.0 nm (fig. S2). The films’ exceptional crystallinity was corroborated by x-ray diffraction (XRD) analysis (Fig. 2A) and scanning transmission electron microscopy (STEM) imaging (Fig. 2B). The STEM cross-sectional image resolved atomic-scale structure at the FeTe/STO interface, whereas XRD patterns revealed a series of (00*n*) Bragg peaks, satisfying the condition 2dsinθ=nλ, where λ=1.5406 Å is the x-ray wavelength. From the room-temperature XRD and STEM measurements, the average lattice parameters of the FeTe film were estimated to be $a_{\mathrm{FeTe}}\approx b_{\mathrm{FeTe}}\approx 3.84\;Å$ and $c_{\mathrm{FeTe}}\approx 6.26\;Å$. Notably, the FeTe/STO interfacial structure exhibits a distinct SrO-Ti*_x_*O_2_-Ti*_x_*O_2_-Te-Fe-Te stacking sequence, as highlighted in the atomic model superimposed on Fig. 2B. The observation of the double titanium oxide termination at the STO interface is noteworthy as this feature has been extensively characterized in the analogous FeSe/STO interface (*45*, *46*), but there have been no reports to date in the FeTe/STO system. Figure 2C shows an atomically resolved scanning tunneling microscopy (STM) topography of the FeTe film. In contrast to cleaved bulk FeTe (*43*, *44*), our film exhibits a defect-free surface without excess Fe atoms. Using a spin-polarized tip, the STM image reveals a striped magnetic contrast with a spacing of 2$a_{\mathrm{FeTe}}$ (Fig. 2D), consistent with the BCAF ordering observed in FeTe with low excess Fe content (*43*, *44*). At low temperature, the atomically resolved STM image shows $a_{\mathrm{FeTe}}/b_{\mathrm{FeTe}}\approx 1.01$, consistent with a low-temperature monoclinic distortion reported for bulk FeTe (*31*, *32*).

**Fig. 2.**
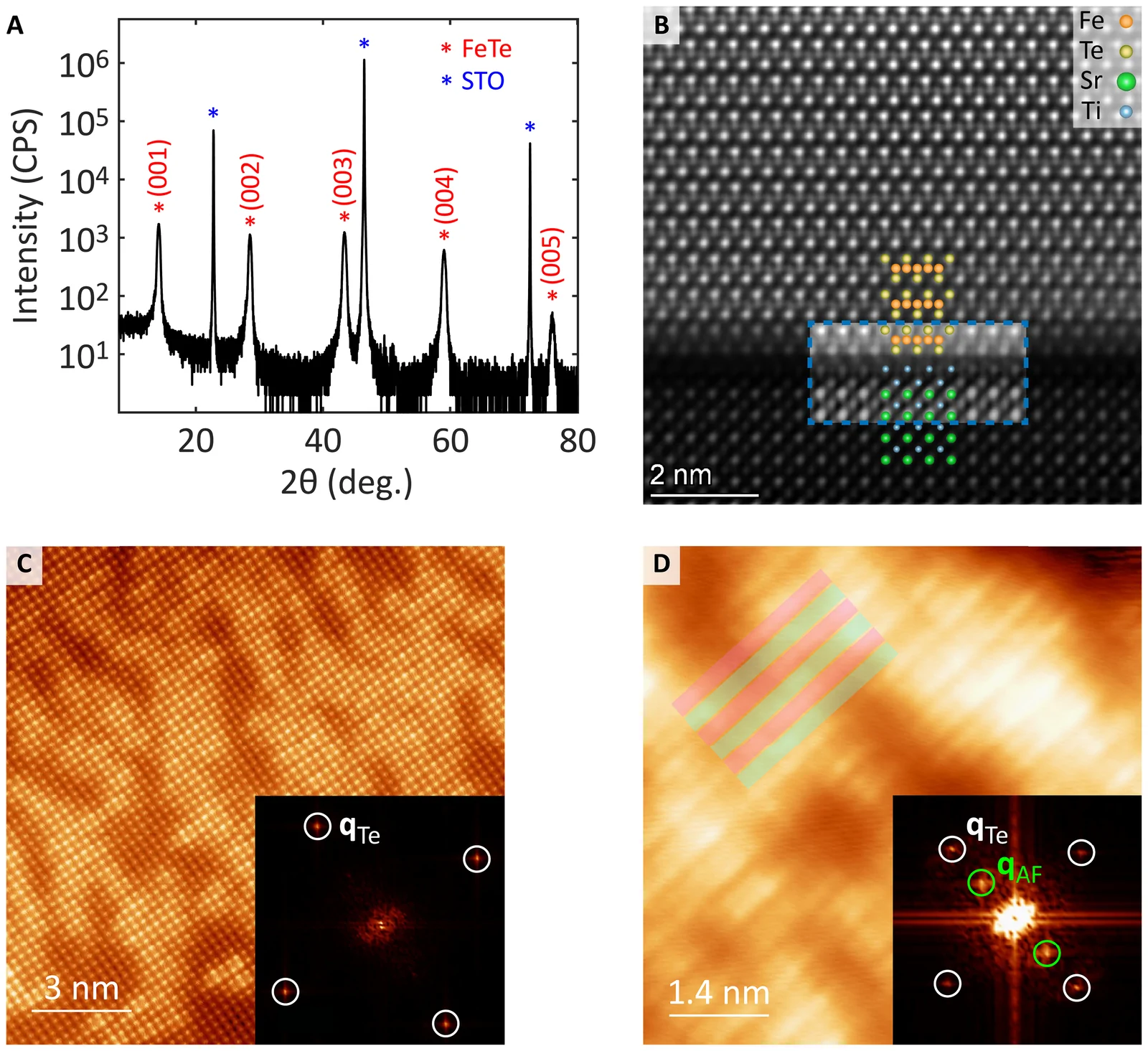
Structural characterizations of the FeTe/STO film. (**A**) XRD patterns of the FeTe/STO film with evident (00*n*) Bragg peaks for both FeTe and STO. (**B**) Cross-sectional high-angle annular dark-field scanning transmission electron microscopy (STEM) image near the interface between FeTe and STO, viewed along the [010] direction. To clarify the interface structure, the color-coded atomic models are partially superimposed on the STEM data. The region enclosed by the blue dashed box is displayed with enhanced brightness to improve the visibility of the interfacial atomic structure. (**C**) Atomically resolved scanning tunneling microscopy (STM) topography of a FeTe film (*V* = −100 mV, *I* = 100 pA). Inset: Fast Fourier transform (FFT) patterns of the topographic image. (**D**) STM topography acquired with a spin-polarized tip (*V* = −100 mV, *I* = 200 pA). Both the stripes of 2$a_{\mathrm{FeTe}}$ spacing and the peaks **q**_AF_ = ^1^/_2_ **q**_Te_ in the FFT patterns (inset) indicate the bicollinear AF order. The colored stripes indicate the configuration of bicollinear AF order.

### AHC in FeTe

Electrical measurements disclose the role played by the BCAF order in FeTe. Longitudinal resistivity (ρ*_xx_*) measurements using a four-probe van der Pauw (vdP) method (see Materials and Methods and fig. S4, A to C) reveal a pronounced maximum at 60 K (Fig. 3A), which is a clear signature of the phase transition from paramagnetic (PM) to AF order at *T*_N_. The magnetic order transition is accompanied by a structural phase transition from tetragonal to monoclinic which stabilizes the BCAF order (*31*, *32*). The peak in the ρ*_xx_*-*T* curve is likely driven by enhanced spin fluctuations, as observed for other AF orderings (*47*, *48*). Above *T*_N_, ρ*_xx_* exhibits a logarithmic temperature dependence

$$\rho_{xx}\left(T\right)=a+b\cdot \text{ln}\left(T\right)$$ (1)

**Fig. 3.**
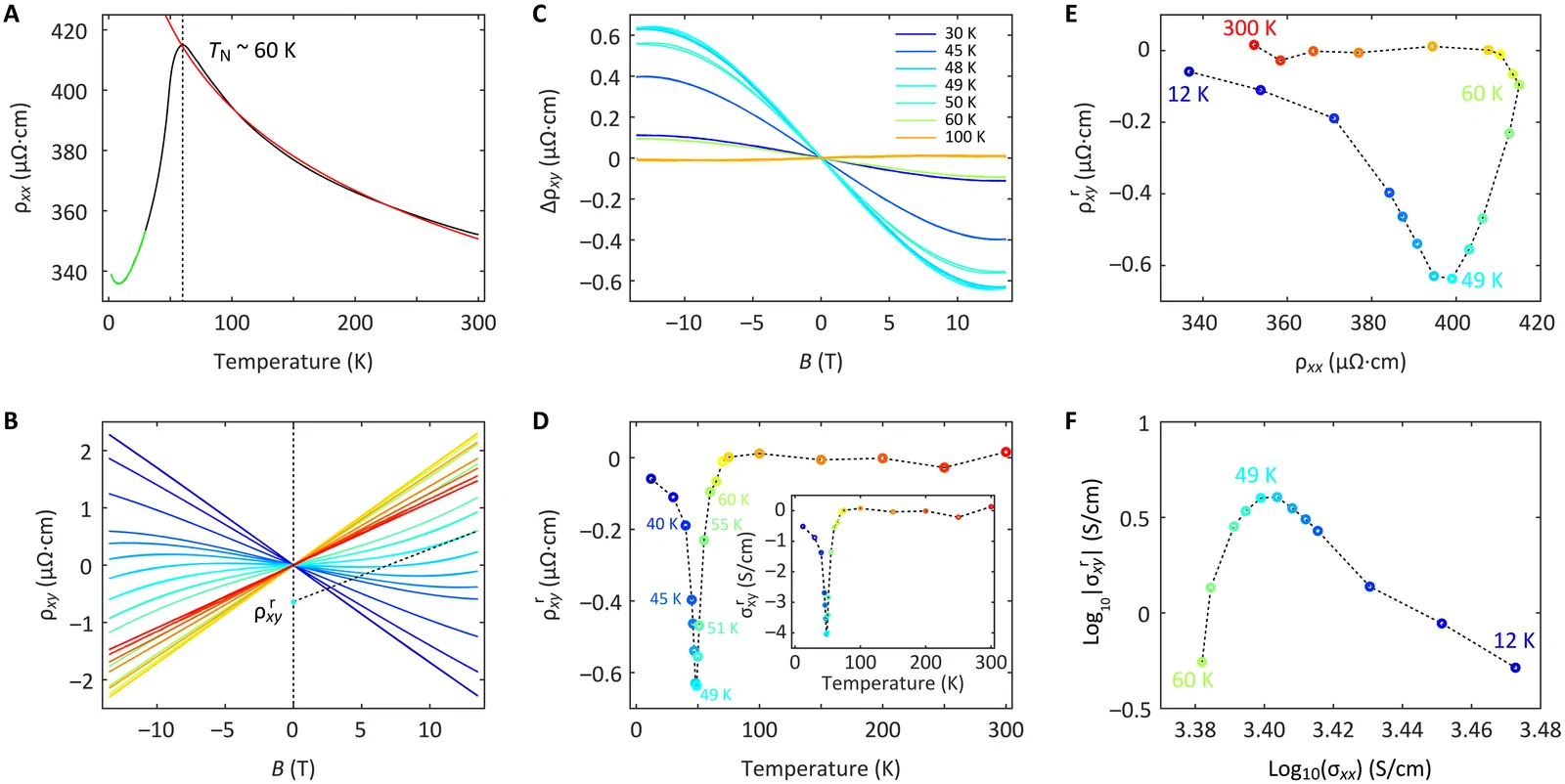
Transport properties and AHE in FeTe/STO. (**A**) Longitudinal resistivity ($\rho_{\mathrm{xx}}$) of the FeTe/STO film. The Néel temperature of the AF transition is identified as 60 K (vertical dashed line). The green curve represents a fit to the ρ*_xx_*(*T*) data for *T* < 30 K using the equation $\rho_{\mathrm{xx}}\left(T\right)=a_{1}+b_{1}\cdot T^{2}+c_{1}\cdot \text{ln}\left(T\right)+d_{1}\cdot T^{5}$, whereas the red curve shows a fit for *T* > 65 K with $\rho_{\mathrm{xx}}\left(T\right)=a_{2}+b_{2}\cdot \text{ln}\left(T\right)$. (**B**) Transverse resistivity ($\rho_{\mathrm{xy}}$) as a function of *B*, measured at temperatures ranging from 12 to 300 K. The temperatures are coded with colors, as indicated in (**D**). The slanted dashed line demonstrates a linear fit of the $\rho_{\mathrm{xy}}-B$ curve at high fields (*B* > 12.5 T), with the *y* intercept defining the residual Hall resistivity ($\rho_{\mathrm{xy}}^{r}$). (**C**) Field dependence of $∆\rho_{\mathrm{xy}}$ obtained by subtracting the high-field linear background from $\rho_{\mathrm{xy}}\left(B\right)$. (**D**) Temperature dependence of $\rho_{\mathrm{xy}}^{r}$ in FeTe/STO. Each data point is obtained from the linear fit of the corresponding color-coded $\rho_{\mathrm{xy}}-B$ curve in (B). (**E**) Scaling relation between $\rho_{\mathrm{xy}}^{r}$ and $\rho_{\mathrm{xx}}$. (**F**) Logarithm scaling law between the absolute value of residual HC |$\sigma_{\mathrm{xy}}^{r}$| and σ*_xx_*.

(fitting details are provided in fig. S5), consistent with the behavior previously reported in FeTe single crystals (*27*). This logarithmic dependence reflects the interplay between fluctuating local moments and itinerant carriers in the PM regime. Below *T*_N_, ρ*_xx_* decreases with decreasing temperature, exhibiting metallic behavior, indicative of suppressed scattering due to the onset of the AF order and Kondo lattice–like coherence (*27*). Intriguingly, a low-temperature upturn appears below 8 K, persisting to the lowest measurement temperature of 2 K. To elucidate this behavior, the ρ*_xx_*(*T*) data below 30 K were fitted using a model incorporating multiple scattering mechanisms

$$\rho_{xx}\left(T\right)=a+b\cdot T^{2}+c\cdot \text{ln}\left(T\right)+d\cdot T^{5}$$ (2)

Here, the $T^{2}$ term describes Fermi liquid behavior, whereas the $T^{5}$ term accounts for phonon-induced scattering, although its contribution is negligible compared with the other contributions. The ln (T) term below 30 K is attributed to Kondo-like impurity scattering, which can be distinguished from the separate Kondo lattice–related picture proposed for FeTe at a higher temperature scale (*27*).

To explore the AHE in FeTe, transverse resistivity (ρ*_xy_*) measurements in an out-of-plane magnetic field (*B*) up to 13.5 T were performed from 12 to 300 K (Fig. 3B). At low fields (<2 T), ρ*_xy_*(*B*) follows a linear dependence on *B*, resembling an ordinary-Hall-effect (OHE)-like behavior in both the AF and PM states in the literature (*36*, *38*). However, at high magnetic fields, an unexpected Hall response, which is nonproportional to *B*, emerges, becoming particularly prominent in the intermediate temperature range (40 to 55 K). This Hall response $∆\rho_{\mathrm{xy}}$ is extracted by subtracting a linear background fitted to the high-field region (*B* > 12.5 T) of the ρ*_xy_*-*B* curves (Fig. 3C). The intercept of these fitted curves defines the magnitude of the residual Hall resistivity ($\rho_{\mathrm{xy}}^{r}$), which also serves as a measure of the curve’s nonlinearity. As shown in Fig. 3D and its inset, both $\rho_{\mathrm{xy}}^{r}$(*T*) and the corresponding HC, $\sigma_{\mathrm{xy}}^{r}\left(T\right)\approx \rho_{\mathrm{xy}}^{r}\left(T\right)/\rho_{\mathrm{xx}}^{2}\left(T\right)$, exhibit a notable negative peak within a narrow temperature window around 49 K, denoted as *T*_p_. The $\sigma_{\mathrm{xy}}^{r}$ increases by almost 10 times from 12 K to *T*_p_ and then rapidly shrinks to nearly zero beyond *T*_N_. To further elucidate the origin of this peak, we inspect the scaling relation of $\rho_{\mathrm{xy}}^{r}$ versus ρ*_xx_* and the absolute value |$\sigma_{\mathrm{xy}}^{r}$| versus σ*_xx_ = 1*/ρ*_xx_*, respectively. As shown in Fig. 3E, $\rho_{\mathrm{xy}}^{r}$ is negligible and shows no clear correlation with ρ*_xx_* above *T*_N_. Below *T*_N_, $\rho_{\mathrm{xy}}^{r}$ maintains an obvious nonmonotonic scaling relation with ρ*_xx_*, reaching a pronounced minimum near ρ*_xx_* = 400 μΩ·cm, which corresponds to *T*_p_. In a logarithmic scale plot of the |$\sigma_{\mathrm{xy}}^{r}$| versus σ*_xx_* for *T* ≤ *T*_N_ in Fig. 3F, the local slope changes from positive to negative across *T*_p_. Although the positive section might relate to the scattering process, the negative section lacks an intuitive origin. Such a deviation from a simple monotonic conductivity dependence suggests that the Hall response cannot be fully described by a single-mechanism picture and also motivates consideration of additional intrinsic electronic structure effects.

Several possible mechanisms could account for the generation of $\rho_{\mathrm{xy}}^{r}$. We first consider the OHE contributions. Previous Hall studies on FeTe films have analyzed their transport using a reduced two-band Drude picture, underscoring the multiband nature of this material (*36*, *37*, *49*). However, the nonlinearity has not emerged at large fields in these previous works. One plausible reason could be that the mobility (μ*_i_*, *i* = *e* for electrons or *h* for holes) is not sufficiently high, so the OHE contribution remains in the linear regime. To check this in our samples, we extracted the slopes at low field, $R_{H}^{\text{Low}}$, and high field, $R_{H}^{\text{High}}$, as shown in Fig. 4 (A and B). We find that the carrier mobility μ in our films, estimated from $R_{H}^{\text{High}}$ (fig. S4D) with a single-band model, is on the order of 1 cm^2^ V^−1^ s^−1^ and comparable with values found in previous works (*36*, *37*, *49*). Meanwhile, the two-band model fitting with four independent parameters of mobility and carrier density yields μ*_i_* at least one order of magnitude larger, which appears unreasonable (figs. S6 and S7)*.* Therefore, a nonlinear OHE response is not expected in our results. Additional insight is provided by the longitudinal thermoelectric response, i.e., the Seebeck effect that indirectly reflects the carrier types and mobilities. We performed thermoelectric voltage (*V*_th_) measurements using the setup shown in Fig. 4C and compared the results with the nominal Hall coefficient. The transport of carriers under a temperature gradient generates a voltage *V*_th_, whose sign and magnitude reflect the dominant carrier type and its relative density in the FeTe. As displayed in Fig. 4D, the measured *V*_th_ exhibits two distinct sign reversals, signaling carrier-type transitions. The first reversal, from positive to negative around 45 K, marks a switch from electron to hole dominance. This behavior is consistent with the sign change in the $R_{H}^{\text{High}}$ (Fig. 4B). Together, both the low mobility and the thermoelectric data indicate that OHE is expected to remain linear with field. Therefore, the nonlinearities in $\rho_{\mathrm{xy}}^{r}$ of FeTe cannot be explained by multiband OHE and instead point toward the AHE mechanism underlying the observed nonlinear field-dependent Hall response.

**Fig. 4.**
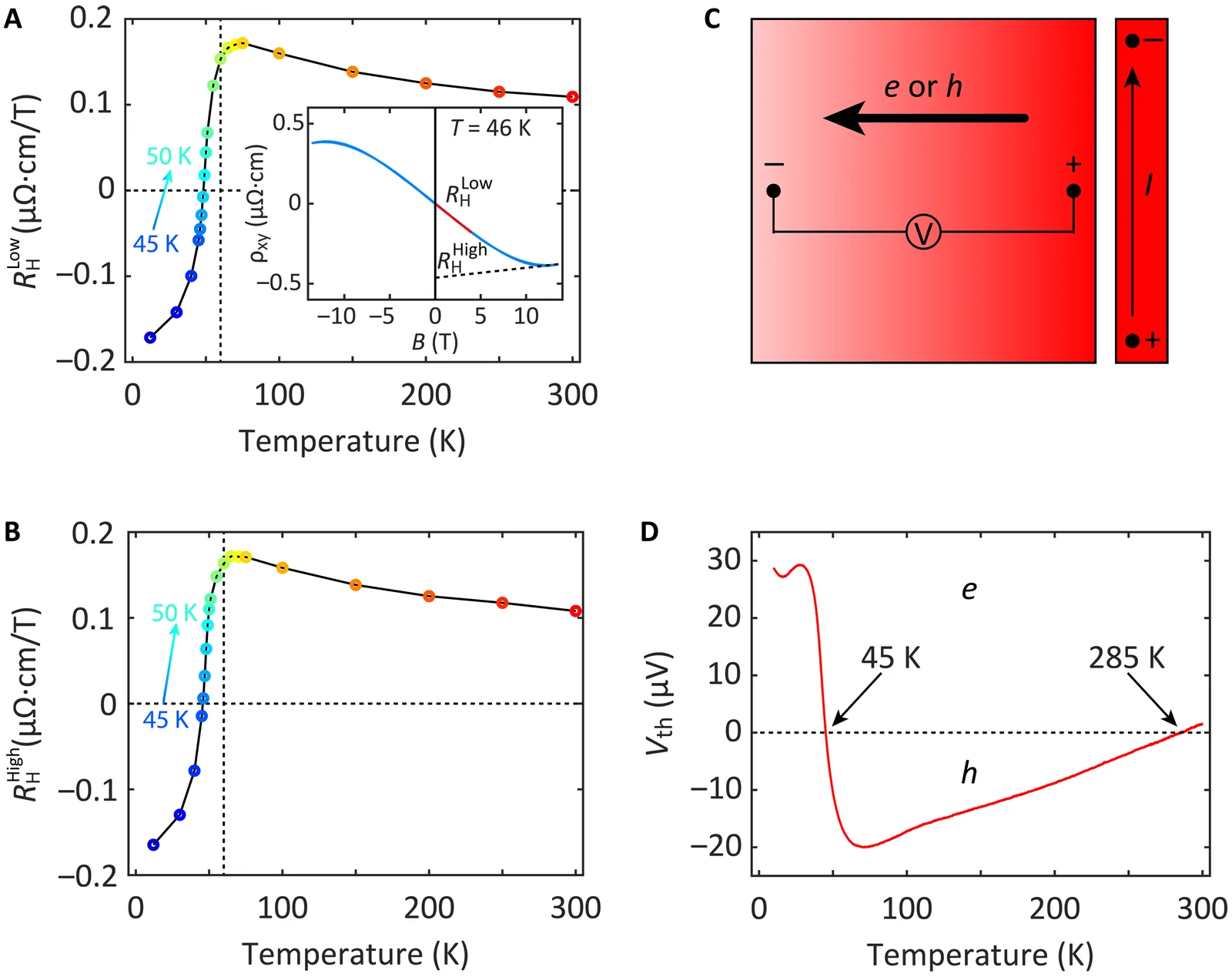
Thermal voltage and Hall coefficients of FeTe/STO. Temperature dependence of the low-field Hall coefficient ($R_{H}^{\text{Low}}$) (**A**) and the high-field Hall coefficient ($R_{H}^{\text{High}}$) (**B**) extracted from the corresponding fitting ranges. Inset: Method for determining the Hall coefficients from the $\rho_{\mathrm{xy}}-B$ curve. $R_{H}^{\text{Low}}$ is extracted through linear fitting in the low-field range (*B* < 4 T), whereas $R_{H}^{\text{High}}$ is obtained from linear fitting in the high-field range (*B* > 12.5 T). (**C**) Schematic diagram of the thermoelectric transport measurement setup. The FeTe film is etched to create an isolated current-heating section (right) and a carrier transport region (left). (**D**) Thermal voltage *V*_th_ of FeTe as a function of temperature. Positive and negative voltage values indicate electron and hole-dominated transport, respectively, with carrier-type transitions at ∼45 and 285 K.

### Tiny magnetic moment in FeTe under an external field

In conventional ferromagnets, an anomalous Hall response often empirically scales with magnetization (*1*–*4*), as described by

$$\rho_{\mathrm{xy}}^{\text{AH}}=R_{s}M_{z}$$ (3)

where *R*_s_ is the AHE coefficient and $M_{z}$ is the out-of-plane component of the magnetization. Therefore, field-induced rotation of the magnetization or changes in the magnetic domain configuration can produce a field dependence of $\rho_{\mathrm{xy}}$ that closely follows that of $M_{z}$. In contrast, AF systems can host more diverse contributions to the AHE (*5*–*16*). Previous reports on FeTe have suggested a correlation between the AHC and the (local) magnetic moment (*30*, *38*). Therefore, it is important to examine whether such a magnetization-related contribution in Eq. 3 plays a dominant role in the AHE of our samples. We measured the field dependence of *M* in our films using a magnetometer (see Materials and Methods). The *M*(*B*) curves, shown in Fig. 5A for an out-of-plane field (*B*//*c*) and in fig. S8 for an in-plane field (*B*//*a*), are dominated by the diamagnetic signal from the STO substrate (fig. S9). The extracted nonlinear contribution (shown in Fig. 5B) reveals a very small *M* with no obvious temperature dependence. The field dependence in Fig. 5B is different from that of ${\Delta \rho }_{\mathrm{xy}}$ (Fig. 3C). The saturation magnetization (*M*_S_) extracted from the high-field regions remains very small across the entire temperature range (Fig. 5C, middle). The contribution from the STO substrate (Fig. 5C, bottom) was measured on a bare substrate and subtracted from the total signal. The isolated contribution from the FeTe film (Fig. 5C, top) shows only a very small magnetic moment without any appreciable temperature dependence unlike that of $\rho_{\mathrm{xy}}^{r}$(*T*) in Fig. 3D. Specifically, $\rho_{\mathrm{xy}}^{r}$ in our case emerges near 49 K, rather than at lower temperatures, and shows no correlation with the measured *M*. We further carried out polar reflective magnetic circular dichroism (RMCD) measurements to probe the field dependence of the *M*_FeTe_ along the out-of-plane direction (Fig. 5D, also see fig. S10). The RMCD signal arises from the volume within the limited skin depth and, therefore, is sensitive to the FeTe moment. The signal varies only linearly with field with a positive slope at each temperature. A clear peak in this slope is observed near *T*_N_ that is characteristic of an AF order. Although not reproducing the field dependence of the $\rho_{\mathrm{xy}}$ or ${\Delta \rho }_{\mathrm{xy}}^{}$, the *M*(*B*) loops indicate small canted magnetic moments with a tilt angle of, at least, about 0.26° (see the Supplementary Materials), which lifts the zero-field symmetry constraint and allows the existence of an AHE.

**Fig. 5.**
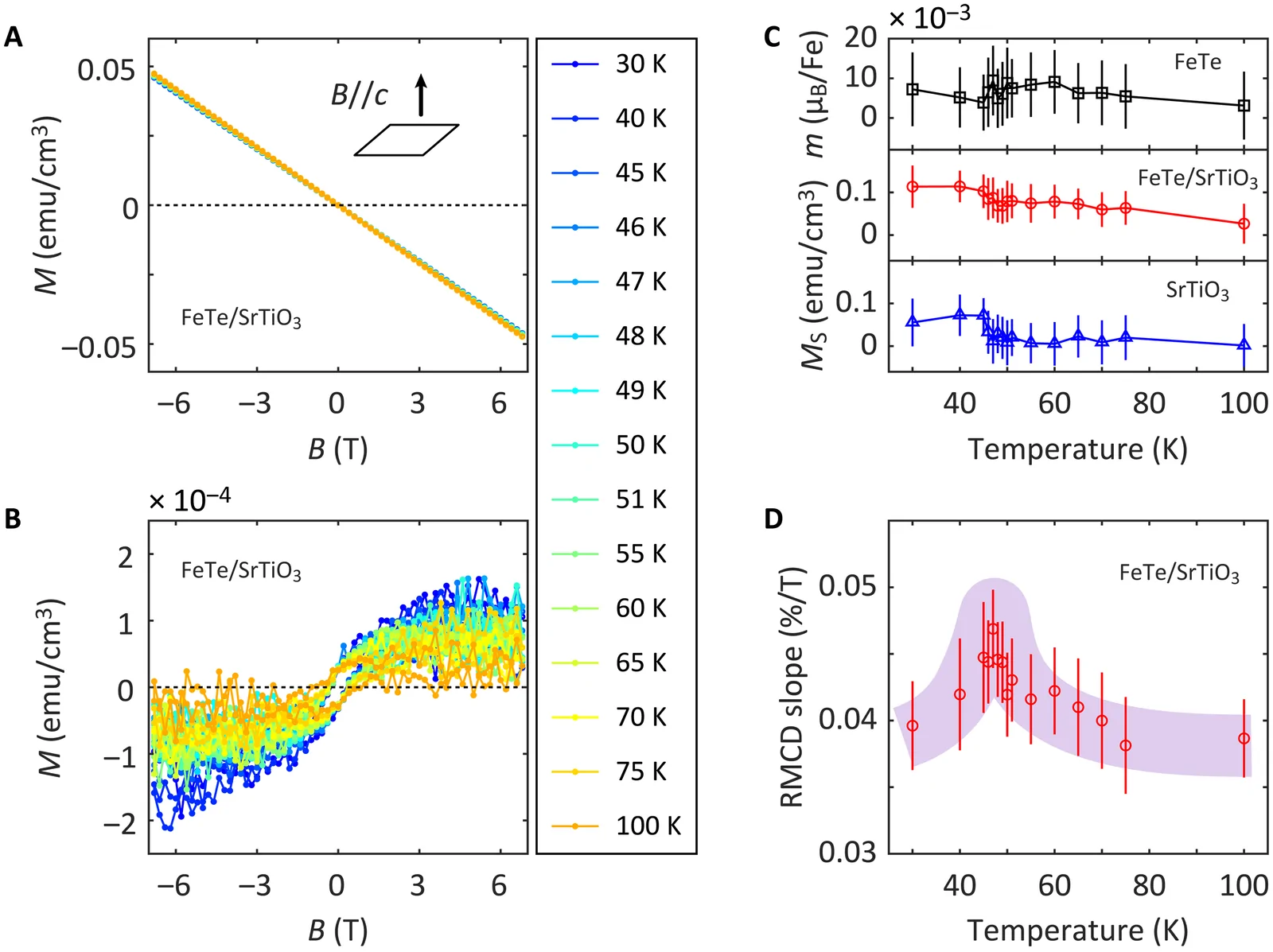
Magnetic moment of FeTe/STO. (**A**) Field-dependent magnetization (*M*) measured by a magnetometer with an out-of-plane magnetic field (*B*//*c*) at various temperatures. (**B**) Magnetization (*M*) versus *B* with a linear background subtracted. The temperature-color correspondence in the legend is shared by (A and B). (**C**) Saturation magnetization (*M*_S_) for *B*//*c* as a function of temperature for the STO substrate (bottom), FeTe/STO (middle), and the difference between them (top), extracted from the intercepts of linear fits to the high-field range of the *M* (*B*) curves. emu, electromagnetic unit. (**D**) Temperature-dependent change in RMCD per unit field for FeTe/STO by linear fitting. The purple shading is a guide to the eye.

### Possible origins of the nonmonotonic scaling

The $\rho_{\mathrm{xy}}^{r}$ likely contains multiple contributions as reported previously (*18*, *20*, *21*) to form the nonmonotonic temperature dependence and scaling behavior, which is also observed in 8-ML and 64-ML FeTe samples (fig. S12). The σ*_xx_* is reduced by about half from 64-ML to 8-ML, and the maximum $\sigma_{\mathrm{xy}}^{r}$ in the 8-ML film is about 0.5 S cm^−1^, accompanied by the disappearance of the positive temperature coefficient of resistance. This $\sigma_{\mathrm{xy}}^{r}$ value is nearly one order of magnitude smaller than those in the 64-ML and 25-ML samples, which does not follow the conventional scaling expected for scattering-driven AHE as $\sigma_{\mathrm{xy}}\propto \sigma_{\mathrm{xx}}$. Meanwhile, the PM-to-BCAF phase transition is present in all three thicknesses and may therefore account for the unconventional AHE scaling. For clarity, we plot the temperature dependence of the first derivative of $\rho_{\mathrm{xx}}$ with respect to *T* for the 25-ML sample, i.e., ${\mathrm{d\rho}}_{\mathrm{xx}}/dT$, in fig. S13A. A peak at 48.3 K indicates the dramatic evolution of the band structure and/or scattering channels, which is, unexpectedly, almost the same as *T*_p_. We show that the $\left|\rho_{xy}^{r}\right|$ exhibits a strong positive correlation with ${\mathrm{d\rho}}_{\mathrm{xx}}/dT$ (fig. S13B). However, a scattering mechanism arising from spin fluctuations, which could result in nonmonotonic *T* dependence, cannot explain the scaling of $\rho_{\mathrm{xy}}^{r}$ in our case as it would rather give rise to a magnitude maximum in the AHC at *T*_N_. Besides, above *T*_N_, transport effects caused by fluctuations usually also have finite intensity, which is absent in our results. This scenario would only be possible if another phase transition or crossover associated with spin fluctuations were identified at *T*_p_. The carrier type change is also not a good candidate as its crossover temperature is around 45 K, smaller than the *T*_p_. The AHE appears as the BCAF builds up, and the negative peak of $\rho_{\mathrm{xy}}^{r}$ takes place at *T*_p_ just below *T*_N_.

Considering a field-induced canting moment in FeTe, a recent theoretical study based on the spin fermion model by Okamoto *et al.* (*35*) has shown that intrinsic Hall response can be generated with a nonzero net Berry curvature. Notably, the calculated σ*_xy_*(*B*) is nearly linear at 0.4 *T*_N_ but becomes distinctly nonlinear around 0.68 *T*_N_ to 0.8 *T*_N_, which is also accompanied by a clear enhancement of the out-of-plane canting moment. This concomitant relationship also exists in our experiment results, namely, that the magnitude of *M_z_* reaches its maximum value between 45 and 50 K, as shown by the RMCD signal (Fig. 5D) around *T*_p_. The calculated temperature dependence of σ*_xy_* by Okamoto *et al.* also indicates a nonmonotonic relationship, whose emergence is sensitive to the Fermi level and tilted magnetization, which is likely the origin of the nonmonotonic scaling relation in this work. For broader context, representative unconventional or generalized AHE scaling behaviors reported in ferromagnetic, ferrimagnetic, and AF systems are summarized in table S1, highlighting that, although deviations from the simplest scaling forms are not unique to FeTe, their microscopic origins differ substantially across material classes.

## DISCUSSION

Compared with representative compensated collinear antiferromagnets (*5*), the Hall resistivity observed in FeTe lies in a comparatively large range. However, it remains at least one order of magnitude smaller than values attributed to intrinsic mechanisms in noncollinear AF materials (*5*) or in some FMs (*1*). This could arise from several factors. While FeTe also has domains with different directions of the BCAF ordering, all domains have, in a nonzero magnetic field, a small net magnetic moment induced along the field direction from spin canting. Thus, contributions from different domains do not cancel each other and the AHC remains robust against variations in the AF domain distribution. However, because the present measurements are performed with the current confined to the (001) plane, we cannot rule out the possibility that FeTe exhibits different AHE responses with current along other directions, especially for current applied perpendicular to the vdW gap. Similar to the case of noncollinear AF ones (*7*, *8*), a single magnetic domain in FeTe may host a spontaneous nonzero AHE when the current is applied along the [001] direction. Meanwhile, spin disorder at magnetic and structural domain walls (*50*) can alter the total AHC. The relationship between the AHE and the magnetic domain structures in FeTe remains to be further investigated as the domain configuration is, in practice, difficult to control. Also, although fluctuation effects cannot account for the AHE origin in FeTe, they could modify the AHC magnitude (*8*, *51*).

Together, the Hall, magnetization, RMCD, and thermoelectric measurements support a coherent picture of a field-enabled intrinsic Hall response in BCAF FeTe. A small field-induced canting breaks the protecting symmetry that enforces Berry-curvature cancellation at zero field; however, its field and temperature dependences do not reproduce those of the nonlinear signal of Hall effects. Consistent with companion theoretical calculations, this decoupling indicates that the induced magnetization acts primarily as a symmetry-breaking switch, whereas the Berry-curvature distribution of the reconstructed bands determines the sign and magnitude of the Hall response. More broadly, these findings suggest that intrinsic Hall transport in compensated AF semimetals can be enabled by breaking the protecting symmetry and can be tuned through band-structure reconstruction near the Fermi level.

## MATERIALS AND METHODS

### Growth of FeTe/SrTiO_3_ films

FeTe films in this work were grown on SrTiO_3_ (STO) (001) substrates using a Veeco GEN10 MBE system under ultrahigh vacuum (UHV) conditions better than 2 × 10^−10^ torr to minimize impurities. Before growth, the STO substrates underwent chemical treatment and high-temperature annealing to achieve an atomically smooth, TiO_2_-terminated surface. The cleaning process involved ultrasonic treatment in acetone and isopropanol to remove adsorbed impurities, followed by immersion in a 90°C hot water bath for 20 min to convert surface SrO into hydroxide complexes. These complexes were then dissolved in a 9 wt % HCl solution for 20 min. After thorough rinsing with deionized water, the substrates were annealed at 1000°C for 3 hours in a tube furnace under an oxygen atmosphere, resulting in well-defined TiO_2_ step-terrace structures. Once transferred to the UHV chamber, they were further degassed at 600°C for 2 hours before film deposition.

FeTe films were synthesized by codepositing Fe (99.99% purity) via a high-temperature Knudsen cell and Te (99.999% purity) via a valved cracker cell, with the substrate temperature maintained at 350°C. Beam flux was characterized using a beam flux monitor, ensuring a Te:Fe flux ratio of ∼9.5:1 and a growth rate of 1 ML/min. Real-time RHEED monitoring (kSA 400) was used to track surface evolution. After deposition, the films were annealed at the growth temperature for 20 min to remove excess Te and enhance crystallinity. Last, a ∼1-nm Al capping layer was deposited in situ after the sample cooled to room temperature. Upon air exposure, this layer naturally oxidized into a dense AlO*_x_* barrier, effectively preventing FeTe oxidation.

X-ray photoelectron spectroscopy (XPS) further indicated the binding energies of Fe and Te remained in their Fe^0^ and Te^0^ states with the protective Al_2_O_3_ capping layer (fig. S3), ruling out surface oxidation or degradation into other impurity compounds (*52*). Unless otherwise specified, all data presented in this work were obtained from FeTe films with a uniform thickness of 25 MLs.

### Structural and magnetic characterization

The structure and quality of the FeTe/STO films were characterized using XRD, atomic force microscopy (AFM), STEM, and STM. XRD measurements were performed on a high-resolution diffractometer (Bruker D8) equipped with monochromatic Cu Kα_1_ radiation (λ = 1.5406 Å), operating at 40 kV and 40 mA. Surface topography was examined by AFM (Bruker Dimension Icon) under ambient conditions. Measurements were conducted in tapping mode with a scan rate of 1 Hz. The acquired images were processed with NanoScope Analysis software using standard flattening procedures. High-angle annular dark-field STEM images were acquired on a spherical-aberration-corrected transmission electron microscope. Atomically resolved surface structure and magnetic ordering were investigated using a UHV STM apparatus (Unisoku USM1600) at 5 K. The FeTe sample for STM measurements was grown on a 0.5 wt % Nb-doped STO substrate using the same recipe as for the transport samples in the Veeco chamber. To prevent surface degradation during the rapid ex situ transfer, a 30-nm-thick Te capping layer was deposited at room temperature and subsequently removed by annealing at 350°C for 30 min in the STM chamber. Topographic images were acquired with a Cr bulk tip, and magnetic contrast was obtained using a spin-polarized tip prepared by gently indenting the tip into the surface to attach a Fe cluster (*53*). The STM images were analyzed by fast Fourier transform using WSxM software. The magnetic moment was determined using a QD MPMS3 as the mentioned magnetometer. RMCD was measured with QD OptiCool.

### Electrical transport measurements

Electrical transport measurements were conducted using a Physical Property Measurement System (QD DynaCool, 1.9 K, 14 T) via the vdP method unless otherwise specified. Four electrodes were manually bonded at the corners of a square sample, as illustrated in fig. S4A, using a wire bonder (West bond). For longitudinal resistivity ($\rho_{\mathrm{xx}}$) measurements, a direct current ($I_{12}=20\;\mathrm{\mu A}$) was applied from electrode 1 to electrode 2 using a Keithley 6221 current source, and the voltage ($U_{43}$, defined as *U*_4_ − *U*_3_) was measured between electrodes 3 and 4 using a Keithley 2182A voltmeter. The $\rho_{\mathrm{xx}}$ was then calculated by averaging the resistances in two orthogonal directions, according to the formula

$$\rho_{\mathrm{xx}}=\frac{\pi }{\text{ln}2}\cdot \frac{U_{43}/I_{12}+U_{23}/I_{14}}{2}\cdot t$$ (4)

where $\frac{\pi }{\text{ln}2}$ is the vdP constant and *t* is the film thickness. The corresponding longitudinal conductivity was determined by $\sigma_{\mathrm{xx}}=1/\rho_{\mathrm{xx}}$.

For Hall effect measurements, a direct current ($I_{13}=100\;\mathrm{\mu A}$) was applied along one diagonal direction of the sample while the voltage ($U_{42}$) was measured along the orthogonal diagonal. The transverse resistivity ($\rho_{\mathrm{xy}}$) was calculated using $\rho_{\mathrm{xy}}=U_{42}/I_{13}\cdot t$. To correct for any asymmetry in electrode placement, the $\rho_{\mathrm{xy}}$ at each magnetic field strength was antisymmetrized using

$$\rho_{\mathrm{xy}}\left(B\right)=\frac{\rho_{\mathrm{xy}}\left(B\right)-\rho_{\mathrm{xy}}\left(-B\right)}{2}$$ (5)

Subsequently, the HC was determined by

$$\sigma_{\mathrm{xy}}\left(B,T\right)\approx \frac{\rho_{\mathrm{xy}}\left(B,T\right)}{\rho_{\mathrm{xx}}{\left(0,T\right)}^{2}}$$ (6)

using the zero-field value of $\rho_{\mathrm{xx}}$, which was found to be negligibly dependent on magnetic field compared with $\rho_{\mathrm{xy}}$ (fig. S4, B and C). All the temperature-dependent measurements were conducted during warming.

## Supplementary Materials

### This PDF file includes:

Supplementary Text Figs. S1 to S13 Table S1 References
