# Supplementary material for: Nonmonotonic scaling of the anomalous Hall effect in a bicollinear antiferromagnet
Source: Sci Adv. 2026 Sep 30;12(40):eaef9859. doi: 10.1126/sciadv.aef9859 (PMC13626080; doi:10.1126/sciadv.aef9859)
Supplement: Supplementary file 1 [file sciadv.aef9859_sm.pdf]

Science Advances
AAAS

Supplementary Materials for

Nonmonotonic scaling of the anomalous Hall effect in a bicollinear antiferromagnet

Ruifeng Wang *et al.*

Corresponding author: Chi Fang, chi.fang@mpi-halle.mpg.de; Stuart S. P. Parkin, stuart.parkin@mpi-halle.mpg.de

Sci. Adv. **12**, eaef9859 (2026)
DOI: 10.1126/sciadv.aef9859

This PDF file includes:

Supplementary Text
Figs. S1 to S13
Table S1
References

Supplementary Text

1. Film characterizations

The FeTe films used in this work were grown using molecular beam epitaxy (MBE) and optimized through a combination of characterization techniques. Under optimized conditions, the Te:Fe flux ratio was maintained at 9.5:1, and the substrate temperature was held at 350°C. Fig. S1 shows the surface characterization from the in-situ reflection high-energy electron diffraction (RHEED) and ex-situ atomic force microscopy. The FeTe films exhibit Volmer-Weber (VW) growth mode, forming well defined tetragonal islands, consistent with previously reported in-situ scanning tunneling microscopy observations (*54*).

Since FeTe films are highly prone to oxidation when exposed to air, AlO_x capping layers were applied to prevent degradation. Previous studies have shown that the Fe^0 and Te^0 in as-grown FeTe rapidly oxidize to Fe^{3+} and a mixture of Te^0/Te^{4+} within minutes of air exposure (*52*, *55*). To verify the effectiveness of our capping method, we performed ex-situ X-ray photoelectron spectroscopy (XPS) using a Thermo Fisher K-Alpha Plus system. Our results showed that the AlO_x-capped FeTe films remained unoxidized even after several months, in stark contrast to earlier reports.

Prior to analysis, the sample surface was cleaned with a 500 eV, 0.5 μA monoatomic Ar^+ ion beam for 30 seconds to remove surface carbon contamination, evidenced by the disappearance of the C 1s peak (Fig. S3A). Throughout the cleaning, the Al 2s and Al 2p peaks remained unchanged, indicating that the AlO_x capping layer was unaffected and remained intact.

Figs. S3, B and C show zoomed-in spectra of the Fe 2p and Te 3d regions, respectively, measured through the thin AlO_x layer. The observed binding energies confirm the presence of Fe^0 and Te^0, with no shifts indicative of oxidation. These results demonstrate that the AlO_x capping layer effectively preserves the intrinsic chemical state of the FeTe film by preventing oxidation.

2. Two band model fitting

In the standard isotropic two-carrier Drude model with electron-like and hole-like channels (*56*), the longitudinal and transverse conductivities are written as

$$\sigma_{xx}(B) = e \sum_{i=e,h} \frac{n_i \mu_i}{1 + (\mu_i B)^2},$$

$$\sigma_{xy}(B) = e \left[\frac{n_h \mu_h^2 B}{1 + (\mu_h B)^2} - \frac{n_e \mu_e^2 B}{1 + (\mu_e B)^2} \right],$$

where n_e and n_h are the carrier densities and μ_e and μ_h are the mobilities of the electron-like and hole-like carriers, respectively. So, only when at least one of the $\mu_i B$ is comparable with 1, the non-linearity becomes obvious. Otherwise, when both $\mu_i B \ll 1$, $\sigma_{xy}(B) \approx e[n_h\mu_h^2 - n_e\mu_e^2]B$, giving the linear B dependence at the same temperature. $\rho_{xy}(B) = -\frac{\sigma_{xy}(B)}{\sigma_{xx}^2(B)+\sigma_{xy}^2(B)}$ also becomes B-linear. This is the reason we mentioned in our main text that $\mu_i B$ is not large enough to give the non-linearity in the FeTe case. We performed the two-band model fitting to further demonstrate this as follows.

The fitting results are shown in Fig. S6, and the corresponding extracted fitting parameters (n_e, n_h, μ_e, and μ_h) are summarized in Fig. S7. The fitting could reproduce the observed ρ_{xy}-B curves well with the four independent parameters. Near 50 K, where the ρ_{xy}^r emerges, at least one result μ_i approaches or exceeds 100 cm^2/(V·s), giving a $\mu_i B \geq 0.1$ T·m^2/(V·s) for $B \geq 10\ T$. The μ_i values, however, are at least one order larger than the literature ones (*36*, *37*, *49*). In other words, if μ_i in the literature are reasonable, a B field in the order of 100 T is expected to create obvious non-linearity reported in this work. This could be the reason that the non-linearity in field dependence of Hall effect has not been observed at large field.

3. Magnetization characterizations

To investigate the magnetic properties of FeTe, we measured the magnetic moment of a FeTe/STO sample and a bare STO substrate using a QD MPMS3 magnetometer. The two samples were cut to similar dimensions (approximately 2.5 mm×5.0 mm). Minor differences in sample size were calibrated using the measured sample mass, enabling us to isolate the magnetic response of the FeTe film by subtracting the corresponding data from the bare STO substrate. Out-of-plane (OOP) magnetization (M) measurements were conducted with the samples mounted in a straw, while in-plane (IP) magnetization measurements were performed with the samples mounted on a quartz rod. All the data (Fig. 5A, Fig. S8 A and C) exhibit an approximately linear behavior, primarily due to the diamagnetic response of the STO substrate. To extract the saturation magnetization (Ms), we fitted the $M(B)$ curves beyond ±3 T for both field-decreasing and field-increasing branches. The Ms values were obtained by averaging the absolute values of the intercepts from the four fits. The temperature dependence of Ms for the pure FeTe film was determined by subtracting Ms(T) curve of the bare STO from that of the FeTe/STO sample. The magnetic field strength (H) was calculated using the relation $H = \frac{B}{\mu_0} - M$. Given the near-linear dependence of M on H, the magnetic susceptibility (χ) was extracted from the slopes of line fits to the $M(H)$ curves.

As a complementary method, we characterized the same FeTe sample using polar reflective magnetic circular dichroism (RMCD), whose signal is sensitive to the OOP magnetic response and serves as a comparative probe of the evolution of magnetic order. This technique thus provides an independent probe of the OOP magnetization. The RMCD signal exhibits a linear dependence on the applied magnetic field (Fig. S10). By performing linear fits of the data in the field range of 1-2 T, we obtained the RMCD slope at each temperature (Fig. 5D), which serves as a characterization of temperature-dependent χ response.

To place the experimentally inferred canting in context, we compared it with the recent spin-fermion calculation by Okamoto et al. (*35*). At 10 T, that calculation gives a field-induced out-of-plane moment of order 0.06-0.09 μ_B/Fe and a canting angle of about 1.5-2.5°, depending on temperature. By contrast, the saturation magnetization measured in our film corresponds to a much smaller but still finite moment of order 0.01 μ_B/Fe, with an estimated canting angle of about 0.26°. We do not interpret this tiny net moment as directly setting the Hall magnitude. Rather, its main significance is that it signals a small but finite field-induced canting capable of lifting the zero-field symmetry constraint that otherwise enforces cancellation of the Berry-curvature contribution.

4. Total Hall conductivity

The σ_{xy} at 10 T (σ_{xy}^{10T}) experiences a crossover from a negative to a positive value at 48 K (Fig. S11A). As the scaling law between σ_{xy} and σ_{xx} shows, σ_{xy}^{r} remains non-zero below T_N and becomes one order of magnitude smaller above T_N (Fig. 3D). In both regions there is no obvious proportional dependence of σ_{xy}^{r} on σ_{xx}. On the other hand, σ_{xy}^{10T} (Fig. S11B) shows a positive correlation with σ_{xx} below the crossover temperature. The linear field-dependent part of σ_{xy} may also contain an intrinsic contribution, which is qualitatively explained by a recent theoretical calculation considering the intrinsic mechanism (*35*).

5. Transport in FeTe with different thicknesses

To distinguish different possible origins of AHE, as well as its non-monotonic scaling relation with longitudinal conductivity σ_{xx}, 8-ML and 64-ML samples (Fig. S12) are also prepared with the same method as the 25-ML sample shown in the main text. The thicker sample gives similar results of T_N about 60 K and temperature dependence of longitudinal resistivity ρ_{xx} (Fig. S12A) or nonlinearity part of Hall resistivity ρ_{xy}^{r}(Fig. S12C and E). The ρ_{xx} of the 64-ML sample exhibits a pronounced anomaly near T_N, showing a metal–semiconductor-like transition. The ρ_{xy}^{r} maintains a peak around 48-49 K and a similar magnitude as the 25-ML sample. In contrast, the 8-ML sample displays a

substantially enhanced ρ_{xx} (Fig. S12B), and its temperature dependence becomes more semiconductor-like with a negative temperature coefficient. Nevertheless, the AF transition remains visible around 50 K in the 8-ML sample with a kink in the ρ_{xx} - T curve, and ρ_{xy}^{r} still develops a peak, with a lower temperature 45 K (Fig. S12E). This indicates that the emergence of the AHE does not rely on the presence of a macroscopic metal–semiconductor transition, but is more closely associated with the AF transition itself, possibly through AF-order-induced band reconstruction.

At the same time, the markedly reduced amplitude of the AHE peak in the 8-ML sample indicates that although AF order is retained, the mechanism responsible for generating the AHE is significantly weakened. This weakening may arise from enhanced interfacial scattering, stronger disorder, carrier localization, and reduced interlayer magnetic coupling in the ultrathin limit. Regarding the AHE mechanisms, this behavior is not consistent with a simple extrinsic-scattering scenario. If the AHE were dominated by conventional skew scattering, one would generally expect ρ_{xy}^{r} to scale positively with ρ_{xx} as scattering increases. Similarly, for a simple side-jump or intrinsic scaling behavior, ρ_{xy}^{r} may increase with ρ_{xx}^{2} under certain conditions. However, in the present case, ρ_{xx} is strongly enhanced in the 8 ML sample, whereas ρ_{xy}^{r} is instead suppressed. This indicates that the weakening of the AHE cannot be explained solely by the increase in longitudinal resistivity. Instead, it more likely reflects the suppression of the effective electronic states that generate Berry curvature or magnetic transverse response under thickness reduction and enhanced disorder.

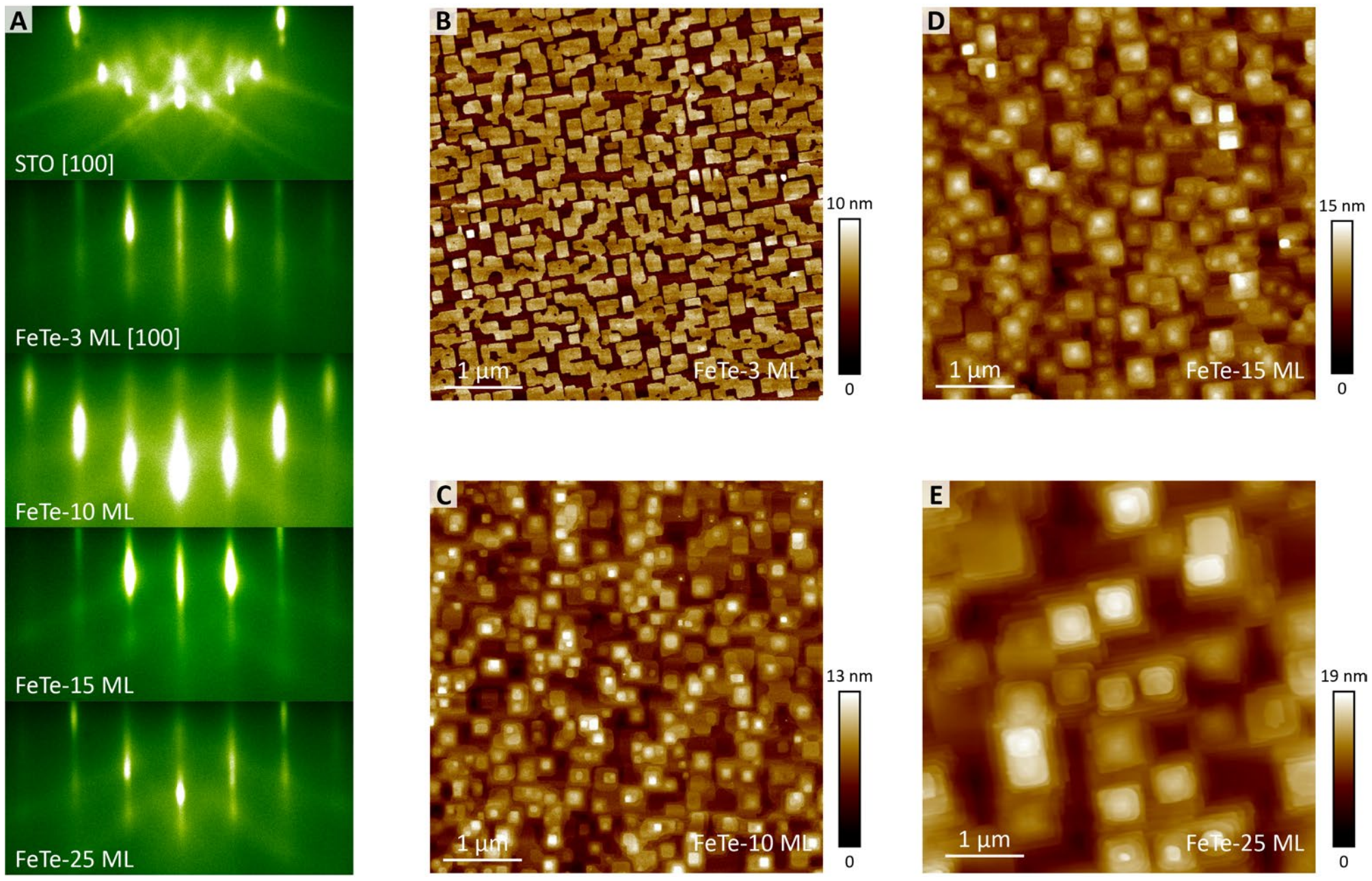

Fig. S1. Topographical characterization of FeTe/STO films with various thicknesses. (**A**) RHEED patterns of a STO (001) substrate and FeTe/STO films with thicknesses of 3 ML, 10 ML, 15 ML, and 25 ML, captured with beam line along the [100] crystallographic direction. Atomic force microscopic images of FeTe/STO films with thicknesses of 3 ML (**B**), 10 ML (**C**), 15 ML (**D**), and 25 ML (**E**).

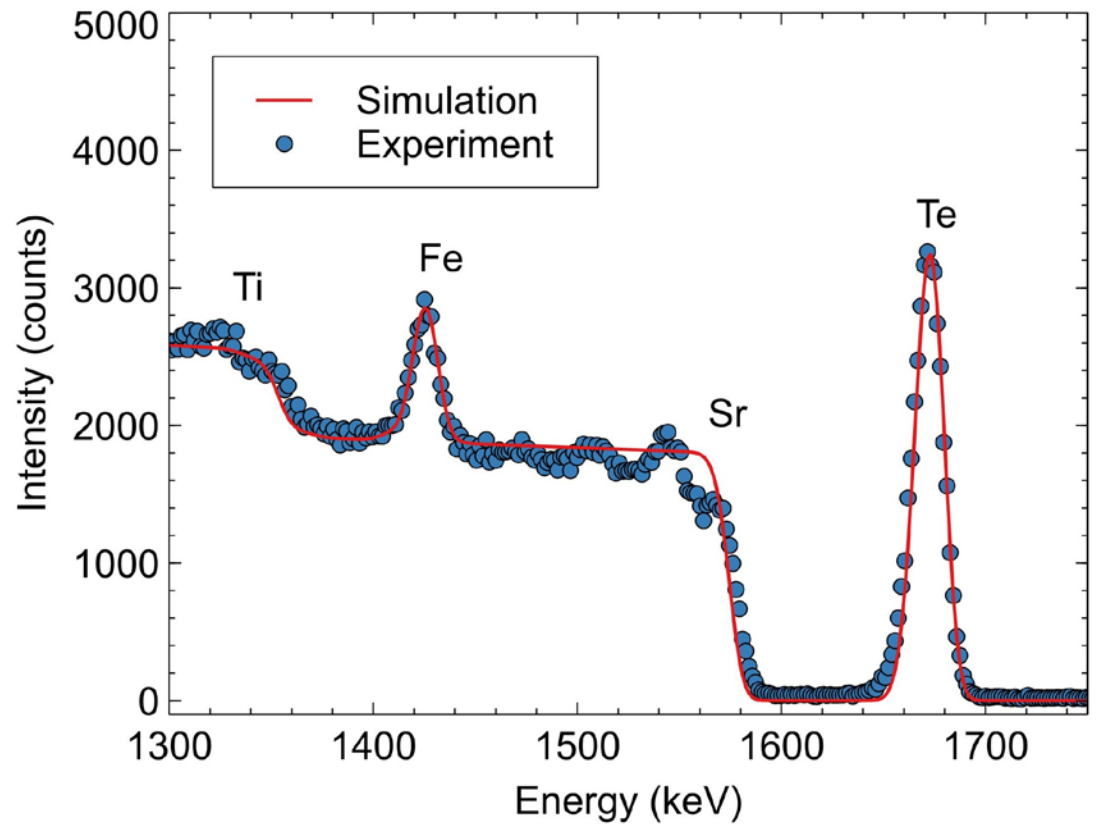

Fig. S2. Rutherford backscattering spectroscopy (RBS) of FeTe/STO. The sample was measured using a He+ 1.9 MeV beam penetrating the surface at -9 deg and collected at a 169 deg scattering angle with a total dose of 20 uQ. Simulation using SimNRA code estimates the atomic surface densities in the units of (10^{15} atoms/cm^2) like following: Fe 36.3 +/- 1 Te 33 +/- 6 corresponding to a composition of $Fe_{1.08}Te$ with a thickness of 15.3+/- 2 nm.

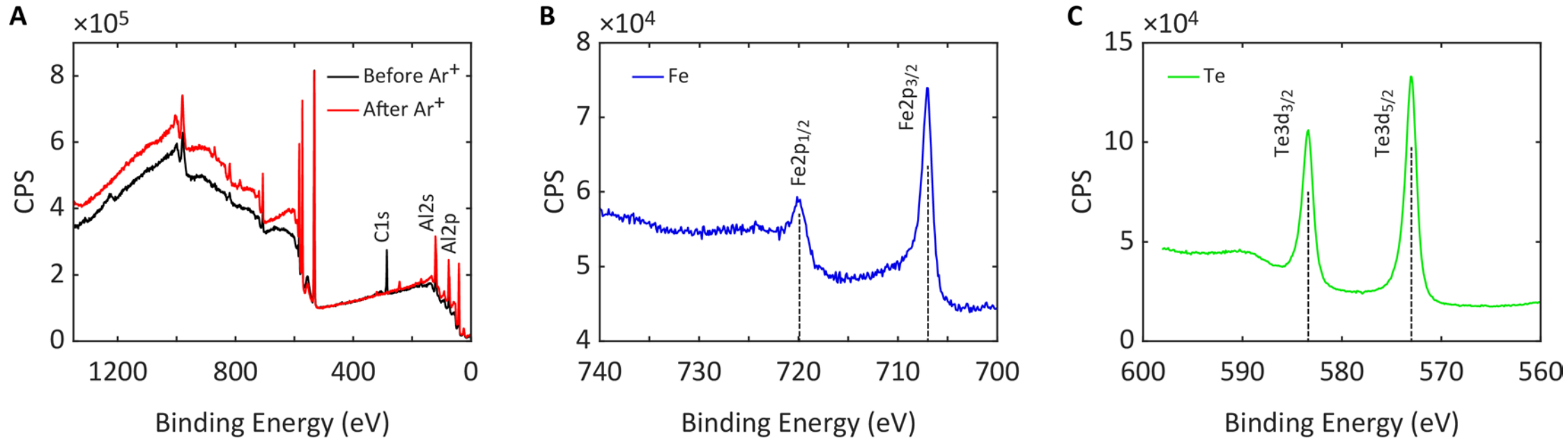

Fig. S3. XPS spectra of FeTe/STO film. (**A**) Wide-range *ex-situ* XPS spectra acquired before and after Ar^+ bombardment of the sample surface. The disappearance of carbon peaks and the persistence of Al peaks confirm the removal of surface contamination while preserving the Al_2O_3 capping. (**B** and **C**) Detailed XPS spectra of Fe-2p and Te-3d regions. The reference binding energies for Fe^0 and Te^0 states are indicated by the dashed lines confirming no oxidation of the film.

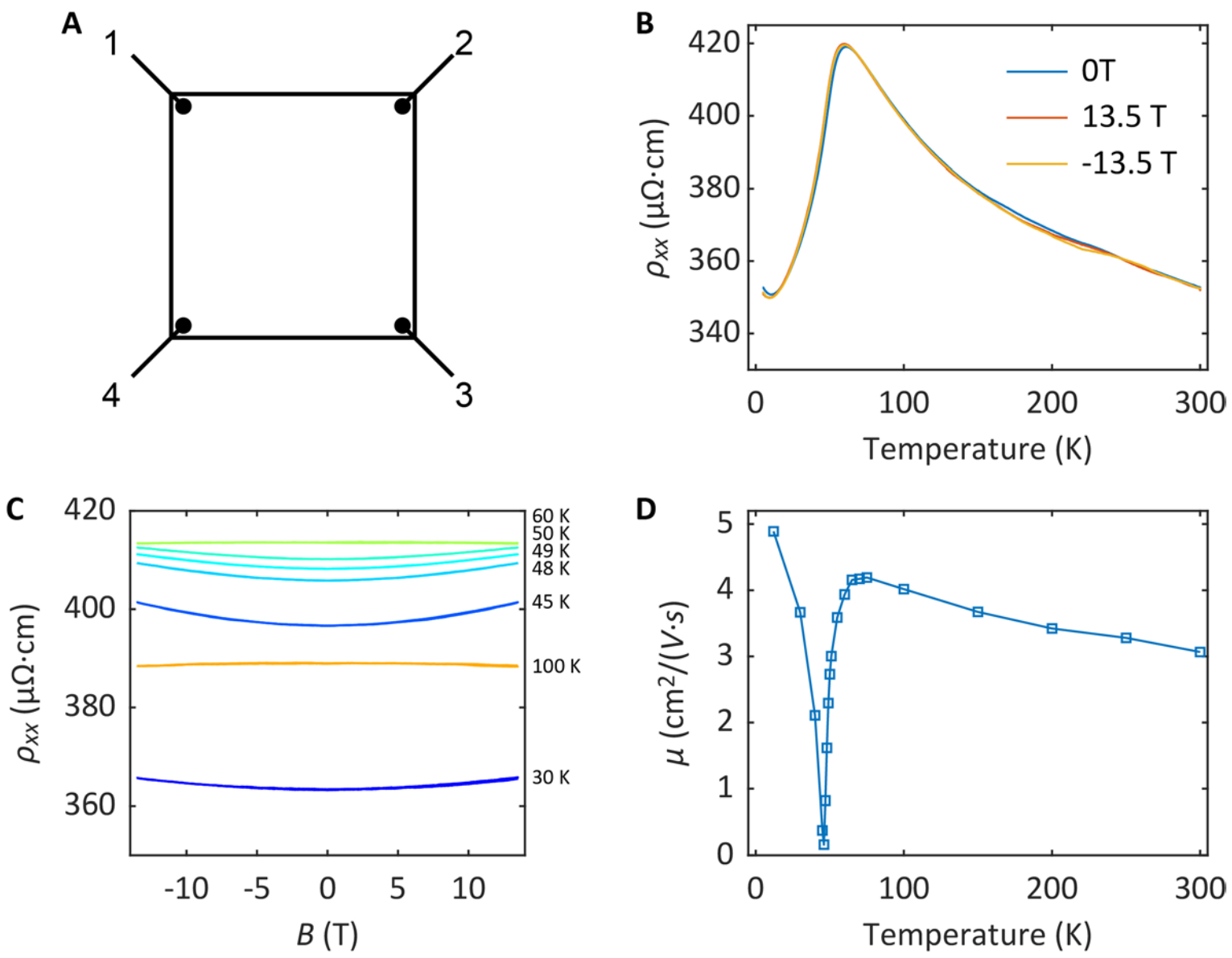

Fig. S4. Longitudinal resistivity and mobility of FeTe/STO. (**A**) Schematic layout of electrodes. (**B**) Comparison of longitudinal resistivity (ρ_{xx}) with and without an applied magnetic field. (**C**) Magnetic field dependence of ρ_{xx} measured at various temperatures. (**D**) Temperature dependent nominal mobility (μ) calculated from R_H^{High} and ρ_{xx}.

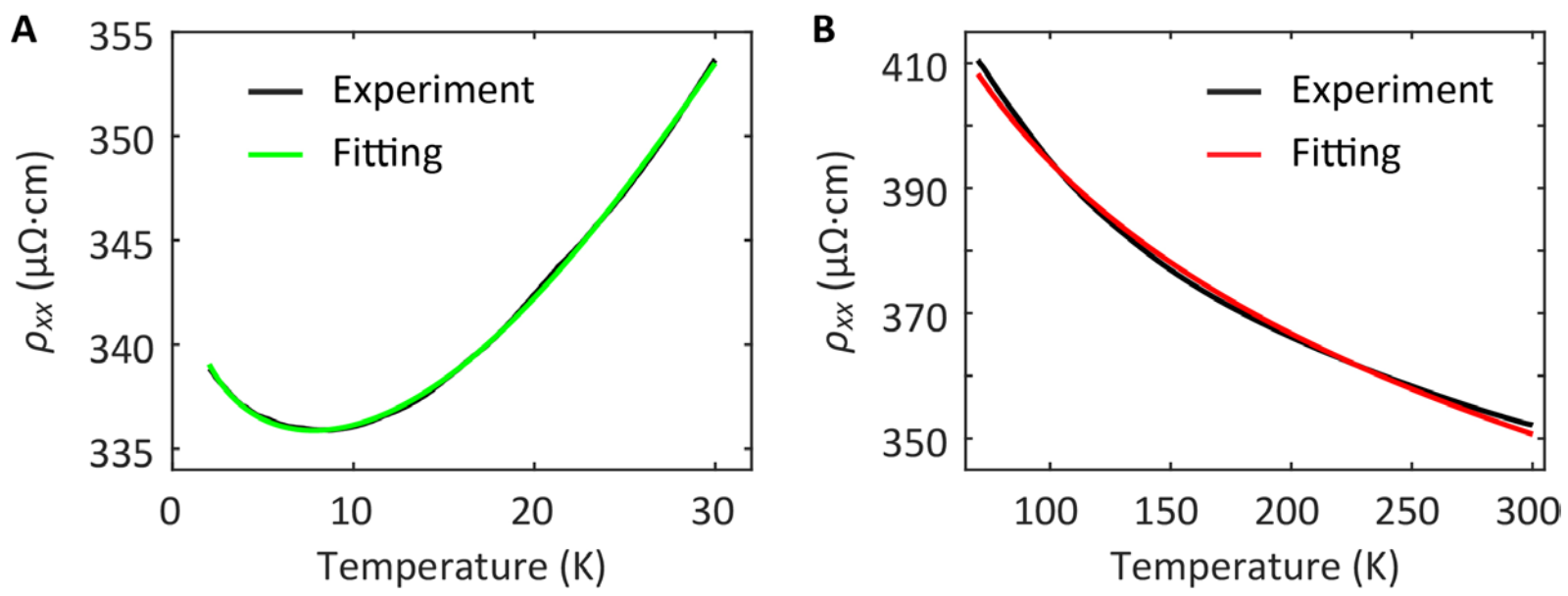

Fig. S5. Fitting details of the temperature dependence of longitudinal resistivity. (**A**) Zoomed-in longitudinal resistivity for T < 30 K and the fitting result using the expression $\rho_{xx}(T) = a_1+b_1T^2+c_1\ln(T)+d_1T^5$. Here, a_1 = 341.5±0.1, b_1 = (2.97±0.03)×10^{-2}, c_1 = -3.61±0.05, d_1 = (-1.00±0.09)×10^{-7}. (**B**) Zoomed-in longitudinal resistivity for T > 65 K and the fitting result using the expression $\rho_{xx}(T) = a_2+b_2\ln(T)$. Here, a_2 = 576.6±0.7, b_2 = -39.6±0.1.

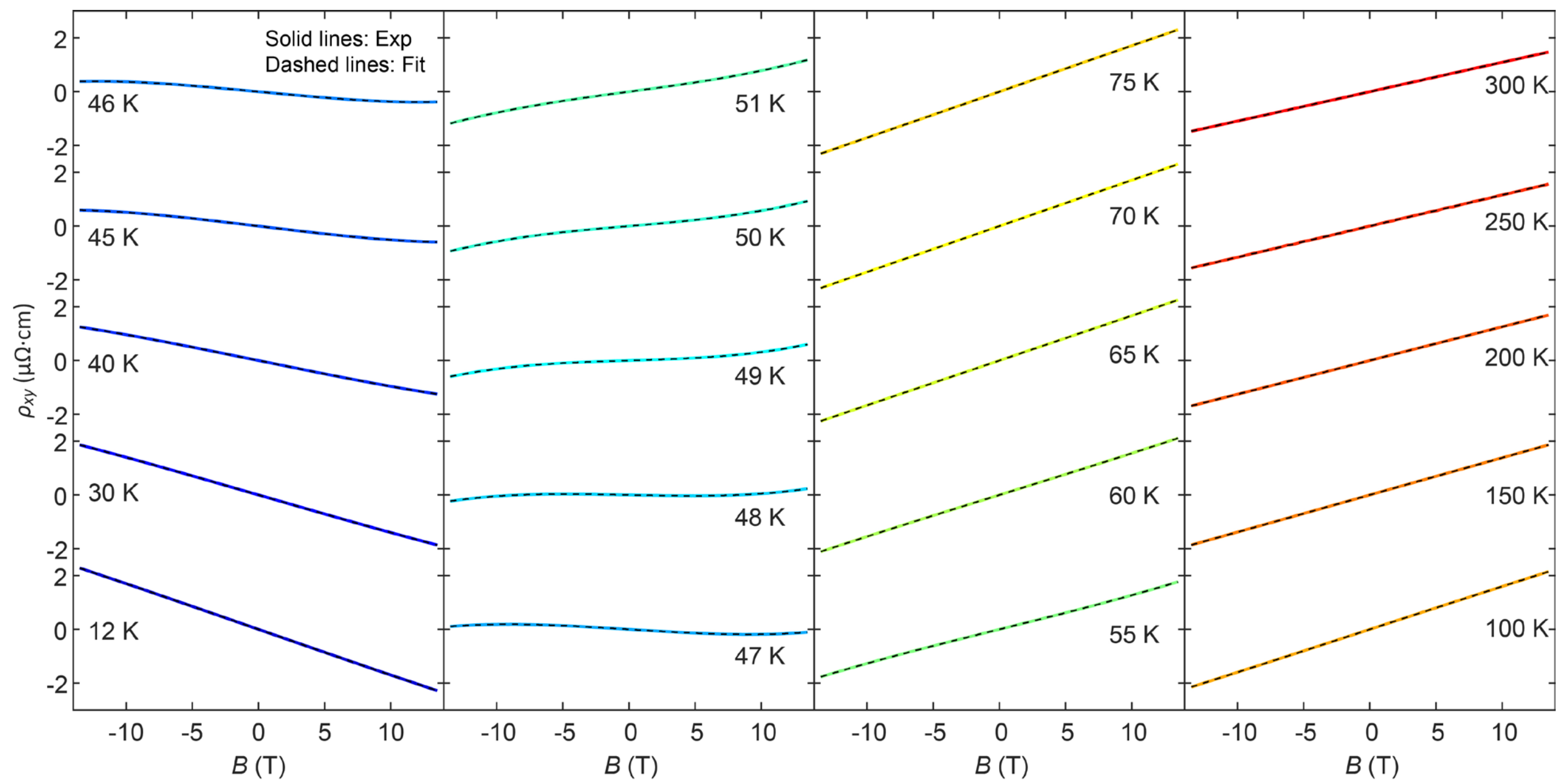

Fig. S6. Experimental Hall resistivity $\rho_{xy}(B)$ of the FeTe film at varied temperatures (solid lines) together with fits using the semiclassical two-band Drude model (dashed lines). Although the fitting reproduces the measured curves well over the full field range, especially near the nonlinear regime around T_N, the extracted transport parameters show strong temperature dependence and are not uniquely constrained by $\rho_{xy}(B)$ alone.

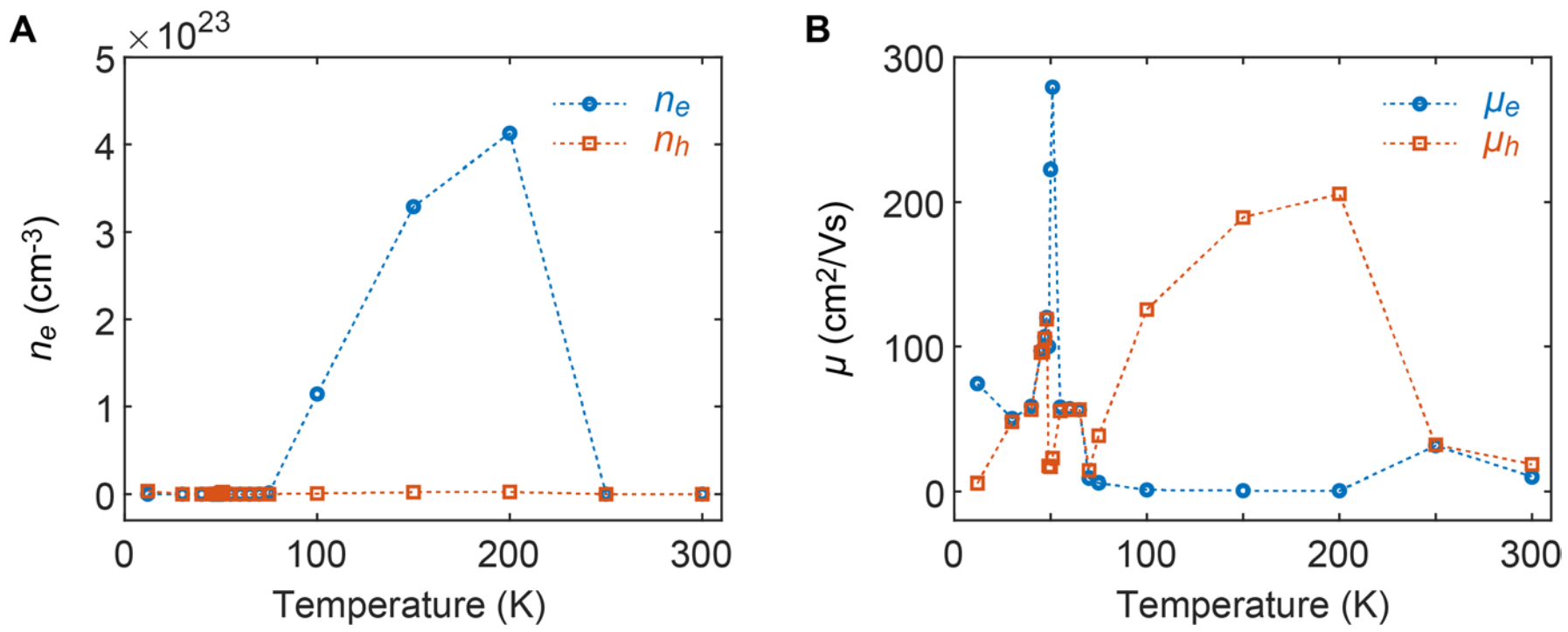

Fig. S7. Temperature-dependent carrier densities and mobilities extracted from the two-band Drude fitting of the FeTe $\rho_{xy}(B)$ data: (**A**) electron and hole carrier densities, n_e, and n_h; (**B**) electron and hole mobilities, μ_e, μ_h. The fitted parameters exhibit strong fluctuations and abrupt changes within a narrow temperature interval, illustrating that good phenomenological fitting does not necessarily imply a unique or physically robust microscopic interpretation.

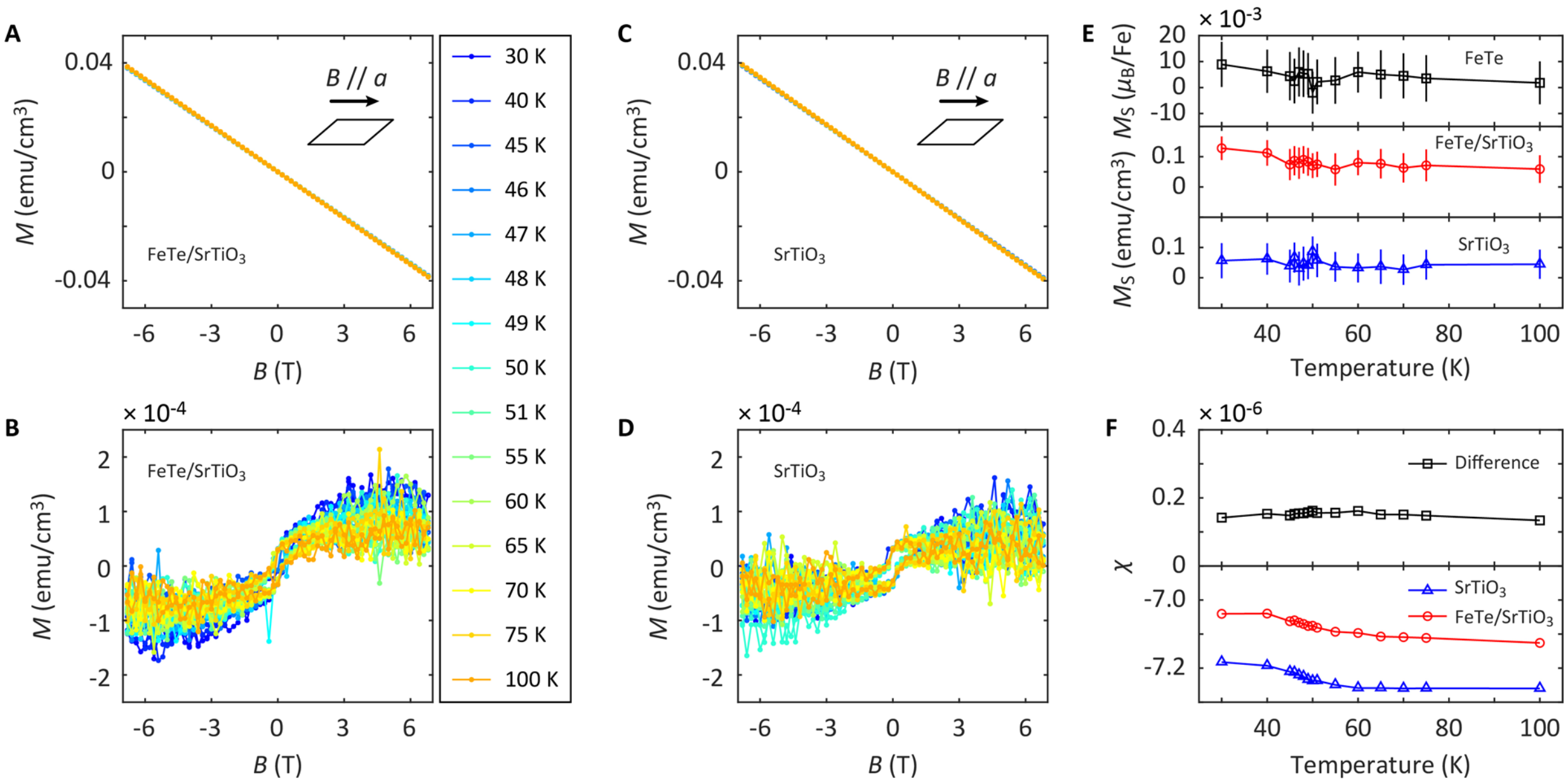

Fig. S8. In-plane magnetization of FeTe/STO. (**A**) Field-dependent magnetization (*M*) of a FeTe/STO sample measured with an in-plane magnetic field (*B* // *a*) at various temperatures. (**B**) Processed data in (**A**), with the linear background subtracted. The temperature-color correspondence in the legend applies to both (**A** and **B**). (**C** and **D**) Corresponding measurements on a bare STO substrate, shown in the same format as (**A** and **B**), respectively. (**E**) Saturation magnetization (*M*s) for *B* // *a* as a function of temperature in the STO substrate (bottom panel), FeTe/STO (middle panel) and the difference between them for pure FeTe (top panel), extracted from the intercepts of linear fits to the high-field ranges of the *M* (*B*) curves, e.g., in (**A**) for FeTe/STO. (**F**) The magnetic susceptibility calculated by linear fitting of the magnetic field dependence of *M* in the FeTe/STO (red circles) and the STO substrate (blue triangles). Black squares show the differences between them.

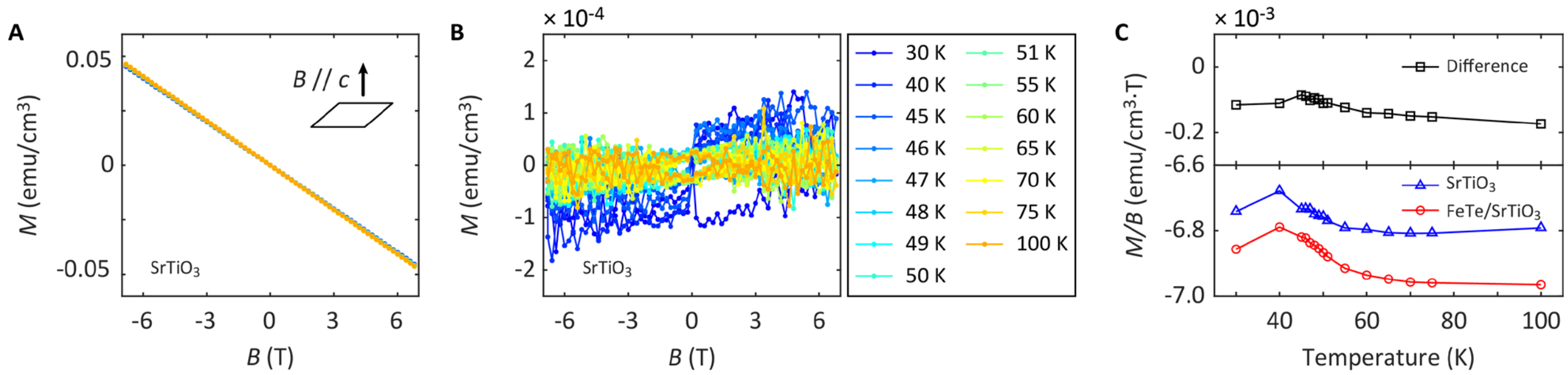

Fig. S9. Out-of-plane magnetization of STO. (**A**) Field-dependent magnetization (*M*) of a STO substrate measured with an OOP magnetic field (*B* // *c*) at various temperatures. (**B**) Processed data in (**A**), with the linear background subtracted. The temperature-color correspondence in the legend applies to both (**A**) and (**B**). (**C**) M/B ratio calculated by linear fitting of magnetic field dependence of *M* above 3 T in FeTe/STO (red circles) and STO substrate (blue triangles). Black squares show the differences between them.

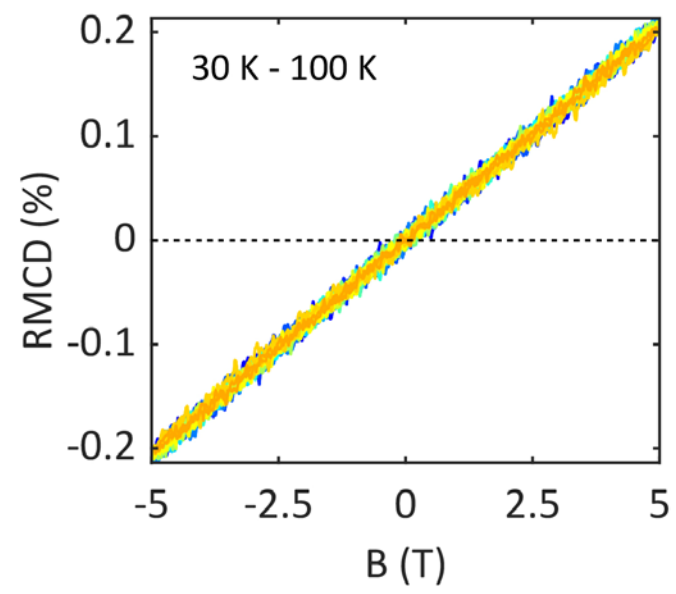

Fig. S10. RMCD signals for FeTe/STO under *B* // *c* at various temperatures.

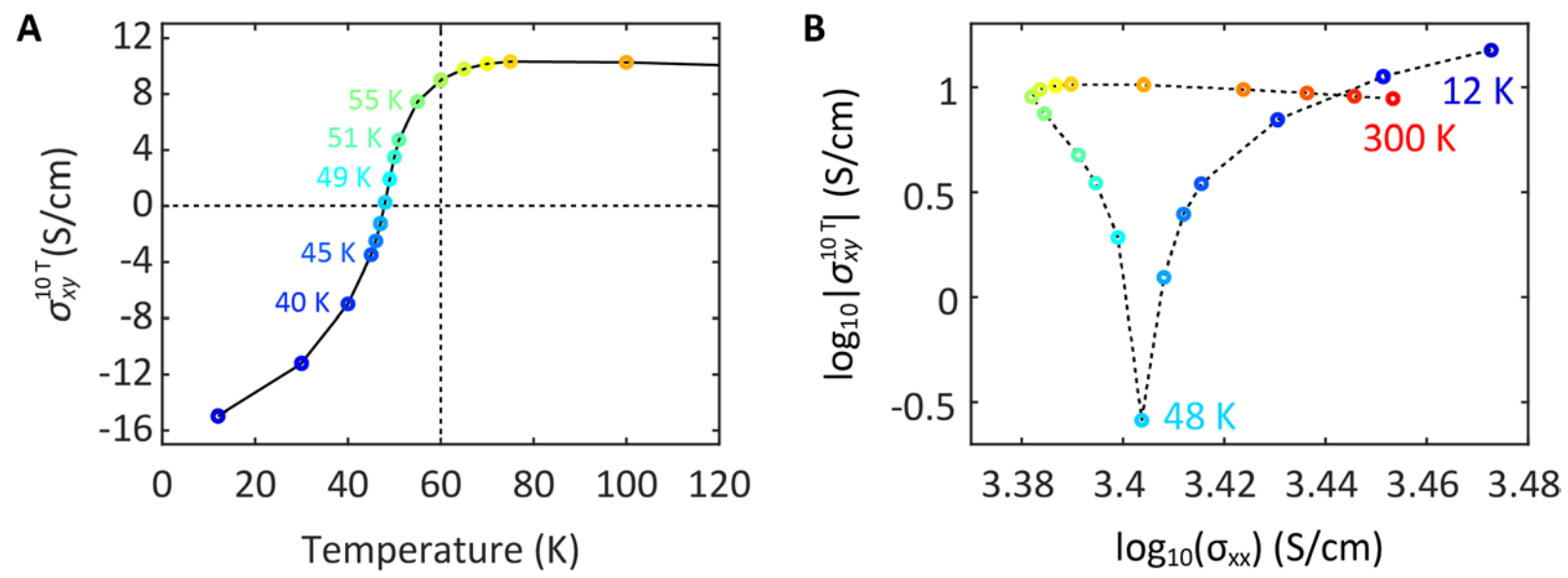

Fig. S11. Logarithm scaling law between Hall conductivity and longitudinal conductivity. (**A**) Experimentally determined temperature-dependent transverse conductivity (σ_{xy}) at 10 T σ_{xy}^{10T}, derived from the ρ_{xx} and ρ_{xy} data in Figs. 3A and 3B. (**B**) Logarithm scaling law between the absolute value of total Hall conductivity at 10 T $|\sigma_{xy}^{10T}|$ and σ_{xx}.

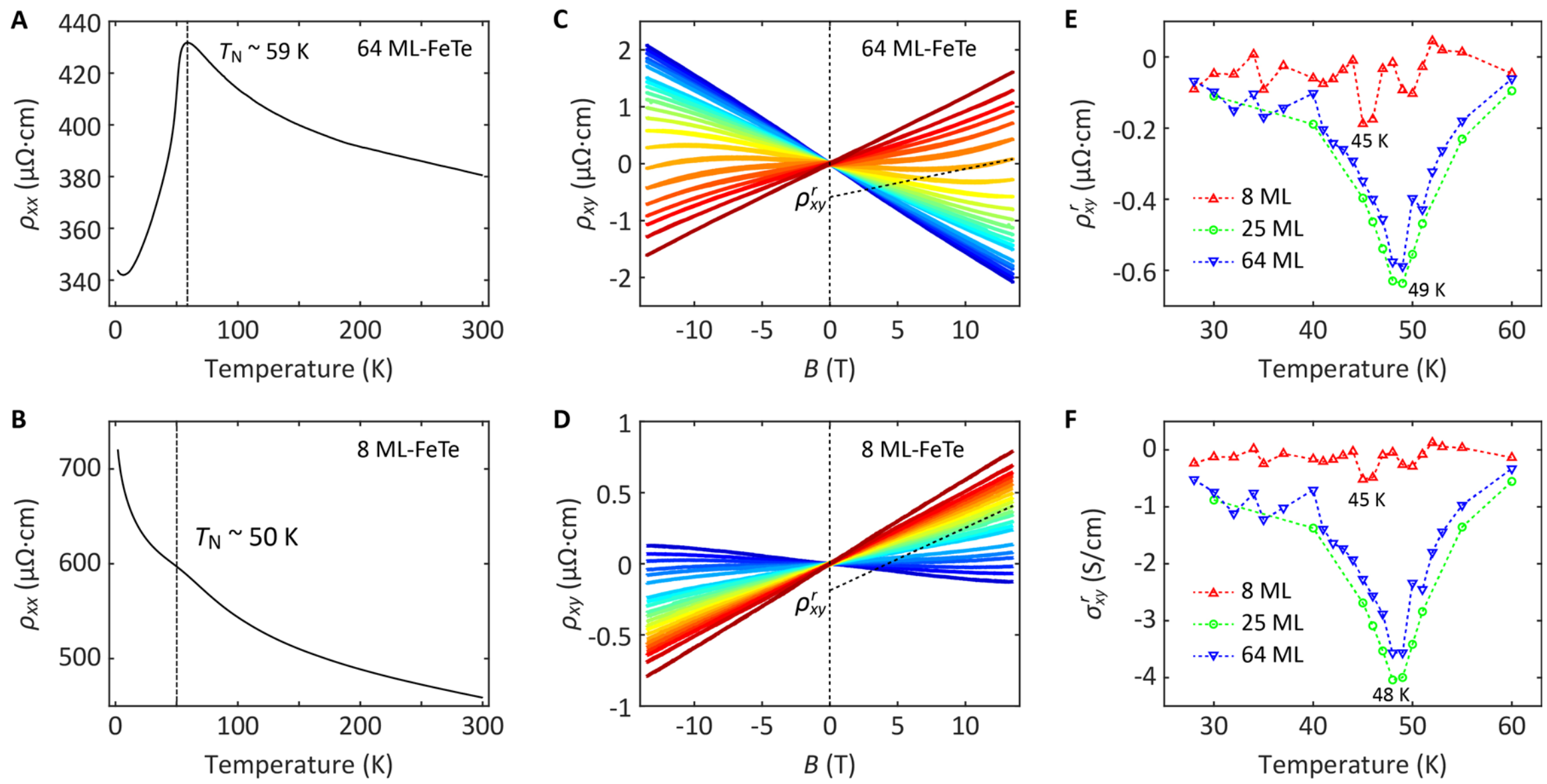

Fig. S12. Transport results of the longitudinal resistivity and Hall response in few-layer FeTe. (**A** and **B**) Temperature dependence of the longitudinal resistivity, ρ_{xx}, for 64-ML and 8-ML FeTe films, respectively. The vertical dashed lines indicate the antiferromagnetic transition temperature, T_N, estimated to be ~59 K for 64-ML FeTe and ~50 K for 8-ML FeTe. (**C** and **D**) Magnetic-field dependence of the Hall resistivity, ρ_{xy}, measured at different temperatures for 64-ML FeTe and 8-ML FeTe, respectively. The dashed lines denote the linear ordinary Hall background, and ρ^r_{xy} represents the extracted nonlinear field-dependent Hall component. **(E)** Temperature dependence of ρ^r_{xy} for FeTe films with thicknesses of 8, 25 (sample in the main text), and 64 ML. A pronounced negative peak emerges near the magnetic transition temperature. (**F**) Corresponding Hall conductivity, σ^r_{xy}, converted from ρ^r_{xy}, showing a maximum response around 48 to 49 K for the 64-ML film, and 45 K for the 8-ML film.

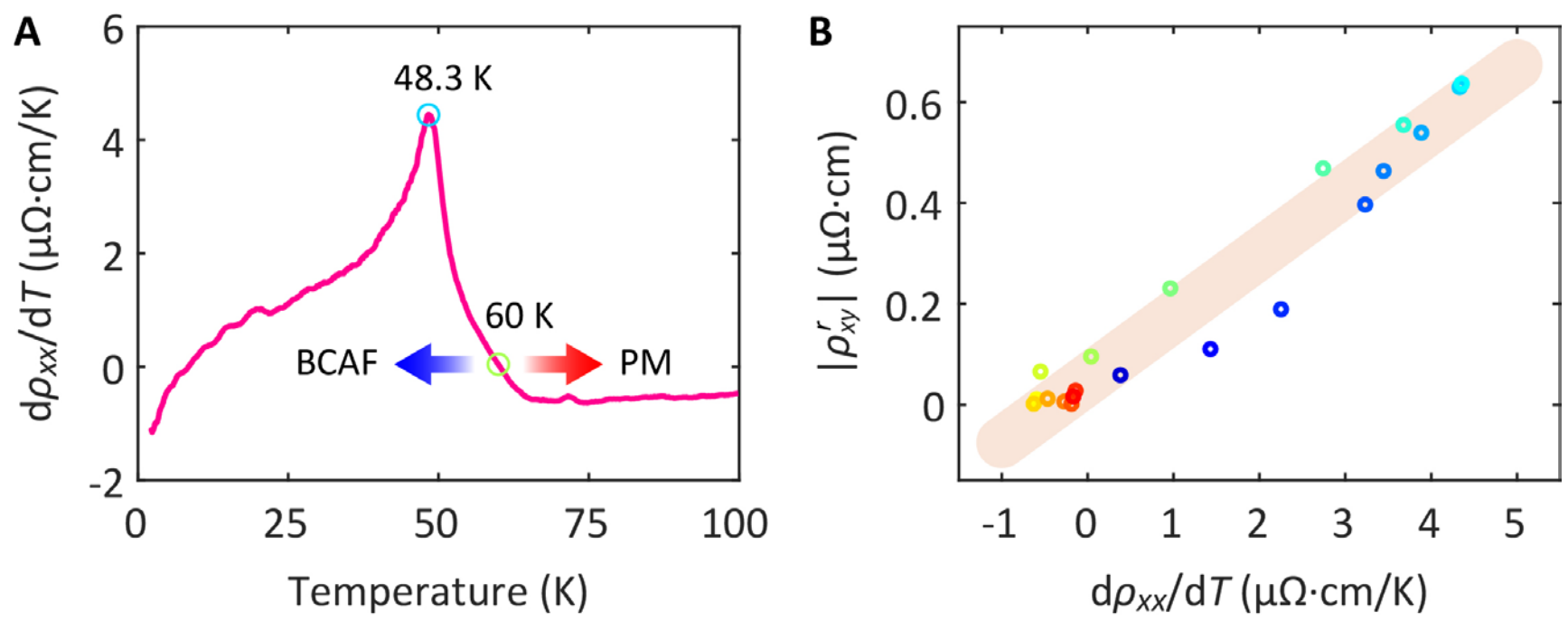

Fig. S13. Correlation between residual Hall resistivity and longitudinal resistivity. (A) Temperature dependence of the first derivative of the longitudinal resistivity, $d\rho_{xx}/dT$. The maximum at 48.3 K coincides with the temperature at which the residual Hall ρ_{xy}^{r} reaches its peak. **(B)** $|\rho_{xy}^{r}|$ plotted as a function of $d\rho_{xx}/dT$ at different temperatures. The data show a linear trend where the shadow is a guide to the eye.

Table S1. Representative unconventional or generalized scaling behaviors reported for anomalous Hall effect in ferro-(FM), ferri-(FiM), and antiferromagnetic (AF) systems.

Material	Magnetic state	Scaling form	Main physical interpretation
Fe, Co, Ni thin films (*17*)	FM	$\rho_{AH} = \alpha_{\mathrm{I}}\rho_{\mathrm{I}}\rho + \beta_{\mathrm{I}}\rho_{\mathrm{I}} + \alpha_{\mathrm{P}}\rho_{\mathrm{P}}\rho + \gamma\rho^2$, the fitting parameters $\alpha_{\mathrm{I,P}}, \beta_{\mathrm{I,P}}, \gamma$ are independent of film thickness or temperature	Surface roughness modifies ρ_{xx} independently of the Hall channel
ε-Fe_3N nanocrystalline films (*19*)	FM	Non-monotonic for amorphous samples	Structural inhomogeneity
USbTe (*18*)	Kondo-lattice FM	Non-monotonic	Kondo hybridization
$TbMn_6Sn_6$ (*20*)	FiM	$\sigma_{xy}^{A} = a\sigma_{xx}^{2} + d/\sigma_{xx} + c$	Magnon related scattering
$Tb(Mn,Cr)_6Sn_6$ (*21*)	FiM	$\sigma_{xy}^{A} = a\sigma_{xx}^{2} + d/\sigma_{xx} + c$	Same as above
Mn_3Pt and related chiral AFs (*14*)	Noncollinear AF	$\rho_{\mathrm{AH}} = a_{sk}\rho_{xx} + b_{in}\rho_{xx}^{2}$	Competition between a scalar spin chirality (SSC)-induced skew scattering topological term a_{sk}, and an intrinsic term b_{in}, whose signs could be opposite.
$PdCrO_2$ (*15*)	Noncollinear AF	σ_{xy} insensitive to σ_{xx}	Spin-cluster skew scattering from fluctuating local scalar spin chirality
$Mn_{2.4}Ga$ (*16*)	Frustrated AF	$\sigma_{xy}^{T} \propto \sigma_{xx}^{2}$	Large Berry curvature from Fermi-level tuning near Weyl points
Present FeTe work	Bicollinear AF	Non-monotonic ρ_{xy}^{r}-ρ_{xx} dependence	Intrinsic contribution combines with band structure evolution near T_{N}

REFERENCES

1. N. Nagaosa, J. Sinova, S. Onoda, A. H. MacDonald, N. P. Ong, Anomalous Hall effect. *Rev. Mod. Phys.* **82**, 1539–1592 (2010).

2. S. Onoda, N. Sugimoto, N. Nagaosa, Intrinsic versus extrinsic anomalous Hall effect in ferromagnets. *Phys. Rev. Lett.* **97**, 126602 (2006).

3. Y. Tian, L. Ye, X. Jin, Proper scaling of the anomalous Hall effect. *Phys. Rev. Lett.* **103**, 087206 (2009).

4. S. Onoda, N. Sugimoto, N. Nagaosa, Quantum transport theory of anomalous electric, thermoelectric, and thermal Hall effects in ferromagnets. *Phys. Rev. B* **77**, 165103 (2008).

5. L. Šmejkal, A. H. MacDonald, J. Sinova, S. Nakatsuji, T. Jungwirth, Anomalous Hall antiferromagnets. *Nat. Rev. Mater.* **7**, 482–496 (2022).

6. R. Shindou, N. Nagaosa, Orbital ferromagnetism and anomalous Hall effect in antiferromagnets on the distorted fcc lattice. *Phys. Rev. Lett.* **87**, 116801 (2001).

7. V. Baltz, A. Manchon, M. Tsoi, T. Moriyama, T. Ono, Y. Tserkovnyak, Antiferromagnetic spintronics. *Rev. Mod. Phys.* **90**, 015005 (2018).

8. A. K. Nayak, J. E. Fischer, Y. Sun, B. Yan, J. Karel, A. C. Komarek, C. Shekhar, N. Kumar, W. Schnelle, J. Kübler, C. Felser, S. S. P. Parkin, Large anomalous Hall effect driven by a nonvanishing Berry curvature in the noncolinear antiferromagnet Mn_3Ge. *Sci. Adv.* **2**, e1501870 (2016).

9. S. Nakatsuji, N. Kiyohara, T. Higo, Large anomalous Hall effect in a non-collinear antiferromagnet at room temperature. *Nature* **527**, 212–215 (2015).

10. X. Li, J. Koo, Z. Zhu, K. Behnia, B. Yan, Field-linear anomalous Hall effect and Berry curvature induced by spin chirality in the kagome antiferromagnet Mn_3Sn. *Nat. Commun.* **14**, 1642 (2023).

11. D. Zhu, J. Lu, Y. Jiang, Z. Zheng, D. Wang, C. Zhou, J. Zhou, S. Chen, Y. Gu, L. Liu, P. Yang, K. Shi, S. Peng, G. Xing, W. Zhao, J. Chen, Observation of anomalous Hall effect in collinear antiferromagnet IrMn. *Nano Lett.* **25**, 4307–4313 (2025).

12. R. D. Gonzalez Betancourt, J. Zubáč, R. Gonzalez-Hernandez, K. Geishendorf, Z. Šobáň, G. Springholz, K. Olejník, L. Šmejkal, J. Sinova, T. Jungwirth, S. T. B. Goennenwein, A. Thomas, H. Reichlová, J. Železný, D. Kriegner, Spontaneous anomalous Hall effect arising from an unconventional compensated magnetic phase in a semiconductor. *Phys. Rev. Lett.* **130**, 036702 (2023).

13. K. P. Kluczyk, K. Gas, M. J. Grzybowski, P. Skupiński, M. A. Borysiewicz, T. Fąs, J. Suffczyński, J. Z. Domagala, K. Grasza, A. Mycielski, M. Baj, K. H. Ahn, K. Výborný, M. Sawicki, M. Gryglas-Borysiewicz, Coexistence of anomalous Hall effect and weak magnetization in a nominally collinear antiferromagnet MnTe. *Phys. Rev. B* **110**, 155201 (2024).

14. S. Xu, B. Dai, Y. Jiang, D. Xiong, H. Cheng, L. Tai, M. Tang, Y. Sun, Y. He, B. Yang, Y. Peng, K. L. Wang, W. Zhao, Universal scaling law for chiral antiferromagnetism. *Nat. Commun.* **15**, 3717 (2024).

15. H. Jeon, H. Seo, J. Seo, Y. H. Kim, E. S. Choi, Y. Jo, H. N. Lee, J. M. Ok, J. S. Kim, Large anomalous Hall conductivity induced by spin chirality fluctuation in an ultraclean frustrated antiferromagnet $PdCrO_2$. *Commun. Phys.* **7**, 162 (2024).

16. L. Song, F. Zhou, H. Li, B. Ding, X. Li, X. Xi, Y. Yao, Y.-C. Lau, W. Wang, Large anomalous Hall effect at room temperature in a Fermi-level-tuned Kagome antiferromagnet. *Adv. Funct. Mater.* **34**, 2316588 (2024).

17. V. L. Grigoryan, J. Xiao, X. Wang, K. Xia, Anomalous Hall effect scaling in ferromagnetic thin films. *Phys. Rev. B* **96**, 144426 (2017).

18. H. Siddiquee, C. Broyles, E. Kotta, S. Liu, S. Peng, T. Kong, B. Kang, Q. Zhu, Y. Lee, L. Ke, H. Weng, J. D. Denlinger, L. A. Wray, S. Ran, Breakdown of the scaling relation of anomalous Hall effect in Kondo lattice ferromagnet USbTe. *Nat. Commun.* **14**, 527 (2023).

19. Y. H. Cheng, R. K. Zheng, H. Liu, Y. Tian, Z. Q. Li, Large extraordinary Hall effect and anomalous scaling relations between the Hall and longitudinal conductivities in ε-Fe_3N nanocrystalline films. *Phys. Rev. B* **80**, 174412 (2009).

20. D. C. Jones, S. Das, H. Bhandari, X. Liu, P. Siegfried, M. P. Ghimire, S. S. Tsirkin, I. I. Mazin, N. J. Ghimire, Origin of spin reorientation and intrinsic anomalous Hall effect in the kagome ferrimagnet $TbMn_6Sn_6$. *Phys. Rev. B* **110**, 115134 (2024).

21. J. M. DeStefano, E. Rosenberg, G. Ren, Y. Lee, Z. Ning, O. Peek, K. Harrison, S. I. Khondaker, L. Ke, I. I. Mazin, J. C. Idrobo, J.-H. Chu, Giant coercivity and enhanced intrinsic anomalous Hall effect at vanishing magnetization in a compensated kagome ferrimagnet. *Sci. Adv.* **11**, eadx4671 (2025).

22. P. Coleman, *Introduction to Many-Body Physics* (Cambridge Univ. Press, 2015).

23. H. Zeng, Y. Zhang, B. Ji, J. Cai, S. Zou, Z. Wang, C. Dong, K. Luo, Y. Yuan, K. Wang, J. Zhang, C. Xi, J. Wang, L. Li, Y. Dai, J. Li, Y. Luo, Kondo-coupled van der Waals antiferromagnet with high-mobility quasiparticles. *Newton* **2**, 100320 (2026).

24. W. Simeth, Z. Wang, E. A. Ghioldi, D. M. Fobes, A. Podlesnyak, N. H. Sung, E. D. Bauer, J. Lass, S. Flury, J. Vonka, D. G. Mazzone, C. Niedermayer, Y. Nomura, R. Arita, C. D. Batista, F. Ronning, M. Janoschek, A microscopic Kondo lattice model for the heavy fermion antiferromagnet $CeIn_3$. *Nat. Commun.* **14**, 8239 (2023).

25. C. Broyles, S. Mardanya, M. Liu, J. Ahn, T. Dinh, G. Alqasseri, J. Garner, Z. Rehfuss, K. Guo, J. Zhu, D. Martinez, D. Li, Y. Hao, H. Cao, M. Boswell, W. Xie, J. G. Philbrick, T. Kong, L. Yang, A. Vishwanath, P. Kim, S.-Y. Xu, J. E. Hoffman, J. D. Denlinger, S. Chowdhury, S. Ran, UOTe: Kondo-interacting topological antiferromagnet in a van der Waals lattice. *Adv. Mater.* **37**, e2414966 (2025).

26. D. Khadka, T. R. Thapaliya, S. Hurtado Parra, X. Han, J. Wen, R. F. Need, P. Khanal, W. Wang, J. Zang, J. M. Kikkawa, L. Wu, S. X. Huang, Kondo physics in antiferromagnetic Weyl semimetal $Mn_{3+x}Sn_{1-x}$ films. *Sci. Adv.* **6**, eabc1977 (2020).

27. Y. Kim, M.-S. Kim, D. Kim, M. Kim, M. Kim, C.-M. Cheng, J. Choi, S. Jung, D. Lu, J. H. Kim, S. Cho, D. Song, D. Oh, L. Yu, Y. J. Choi, H.-D. Kim, J. H. Han, Y. Jo, J. H. Shim, J. Seo, S. Huh, C. Kim, Kondo interaction in FeTe and its potential role in the magnetic order. *Nat. Commun.* **14**, 4145 (2023).

28. I. A. Zaliznyak, Z. Xu, J. M. Tranquada, G. Gu, A. M. Tsvelik, M. B. Stone, Unconventional temperature enhanced magnetism in $Fe_{1.1}Te$. *Phys. Rev. Lett.* **107**, 216403 (2011).

29. R. Liu, Z. Chen, X. Cheng, X. Ren, Y. Zhang, X. Wu, C. Zhang, K. Qian, C. H. Chan, J. Liu, K. T. Law, Q. Shao, Correlation-driven quantum geometry effects in a Kondo system. arXiv:2507.01824 [cond-mat.str-el] (2025).

30. L. Kang, C. Ye, X. Zhao, X. Zhou, J. Hu, Q. Li, D. Liu, C. M. Das, J. Yang, D. Hu, J. Chen, X. Cao, Y. Zhang, M. Xu, J. Di, D. Tian, P. Song, G. Kutty, Q. Zeng, Q. Fu, Y. Deng, J. Zhou, A. Ariando, F. Miao, G. Hong, Y. Huang, S. J. Pennycook, K.-T. Yong, W. Ji, X. Renshaw Wang, Z. Liu, Phase-controllable growth of ultrathin 2D magnetic FeTe crystals. *Nat. Commun.* **11**, 3729 (2020).

31. E. E. Rodriguez, C. Stock, P. Zajdel, K. L. Krycka, C. F. Majkrzak, P. Zavalij, M. A. Green, Magnetic-crystallographic phase diagram of the superconducting parent compound $Fe_{1+}x$Te. *Phys. Rev. B* **84**, 064403 (2011).

32. W. Bao, Y. Qiu, Q. Huang, M. A. Green, P. Zajdel, M. R. Fitzsimmons, M. Zhernenkov, S. Chang, M. Fang, B. Qian, E. K. Vehstedt, J. Yang, H. M. Pham, L. Spinu, Z. Q. Mao, Tunable (δπ, δπ)-type antiferromagnetic order in α-Fe(Te,Se) superconductors. *Phys. Rev. Lett.* **102**, 247001 (2009).

33. M. G. Vergniory, L. Elcoro, C. Felser, N. Regnault, B. A. Bernevig, Z. Wang, A complete catalogue of high-quality topological materials. *Nature* **566**, 480–485 (2019).

34. Topological Materials Database; https://topologicalquantumchemistry.org.

35. S. Okamoto, A. Moreo, N. Nagaosa, S. S. P. Parkin, Berry curvature and field-induced intrinsic anomalous Hall effect in an antiferromagnet FeTe. arXiv:2604.11583 [cond-mat.mtrl-sci] (2026). https://doi.org/10.1038/s42005-026-02751-z.

36. I. Tsukada, M. Hanawa, S. Komiya, A. Ichinose, T. Akiike, Y. Imai, A. Maeda, Mobility analysis of FeTe thin films. *J. Physical Soc. Japan* **80**, 023712 (2011).

37. I. Tsukada, M. Hanawa, S. Komiya, A. Ichinose, T. Akiike, Y. Imai, A. Maeda, Hall effect of FeTe and $Fe(Se_{1-}xTe_{x})$ thin films. *Phys. C Supercond. Appl.* **471**, 625–629 (2011).

38. M. Meng, S. Liu, D. Song, X. Zhang, H. Du, H. Huang, H. Liu, Z. Sun, C. Mei, H. Yang, H. Tian, Y. Lu, Y. Zhang, J. Li, Y. Zhao, Magnetotransport property of oxygen-annealed $Fe_{1+}yTe$ thin films. *J. Phys. Condens. Matter* **35**, 305701 (2023).

39. F. Ma, W. Ji, J. Hu, Z.-Y. Lu, T. Xiang, First-principles calculations of the electronic structure of tetragonal α-FeTe and α-FeSe crystals: Evidence for a bicollinear antiferromagnetic order. *Phys. Rev. Lett.* **102**, 177003 (2009).

40. M. J. Han, S. Y. Savrasov, Doping driven (π, 0) nesting and magnetic properties of $Fe_{1+}xTe$ superconductors. *Phys. Rev. Lett.* **103**, 067001 (2009).

41. S. Rößler, D. Cherian, W. Lorenz, M. Doerr, C. Koz, C. Curfs, Y. Prots, U. K. Rößler, U. Schwarz, S. Elizabeth, S. Wirth, First-order structural transition in the magnetically ordered phase of $Fe_{1.13}Te$. *Phys. Rev. B* **84**, 174506 (2011).

42. C. Koz, S. Rößler, A. A. Tsirlin, S. Wirth, U. Schwarz, Low-temperature phase diagram of $Fe_{1+}yTe$ studied using x-ray diffraction. *Phys. Rev. B* **88**, 094509 (2013).

43. M. Enayat, Z. Sun, U. R. Singh, R. Aluru, S. Schmaus, A. Yaresko, Y. Liu, C. Lin, V. Tsurkan, A. Loidl, J. Deisenhofer, P. Wahl, Real-space imaging of the atomic-scale magnetic structure of $Fe_{1+}yTe$. *Science* **345**, 653–656 (2014).

44. C. Trainer, C. M. Yim, C. Heil, F. Giustino, D. Croitori, V. Tsurkan, A. Loidl, E. E. Rodriguez, C. Stock, P. Wahl, Manipulating surface magnetic order in iron telluride. *Sci. Adv.* **5**, eaav3478 (2019).

45. F. Li, Q. Zhang, C. Tang, C. Liu, J. Shi, C. Nie, G. Zhou, Z. Li, W. Zhang, C.-L. Song, K. He, S. Ji, S. Zhang, L. Gu, L. Wang, X.-C. Ma, Q.-K. Xue, Atomically resolved FeSe/$SrTiO_3$(001) interface structure by scanning transmission electron microscopy. *2D Mater.* **3**, 024002 (2016).

46. H. Sims, D. N. Leonard, A. Y. Birenbaum, Z. Ge, T. Berlijn, L. Li, V. R. Cooper, M. F. Chisholm, S. T. Pantelides, Intrinsic interfacial van der Waals monolayers and their effect on the high-temperature superconductor FeSe/$SrTiO_3$. *Phys. Rev. B* **100**, 144103 (2019).

47. P. E. Brommer, N. H. Duc, "Magnetoelasticity in nanoscale heterogeneous materials," in *Encyclopedia of Materials: Science and Technology*, K. H. J. Buschow, R. W. Cahn, M. C. Flemings, B. Ilschner, E. J. Kramer, S. Mahajan, P. Veyssière, Eds. (Elsevier, Oxford, 2004), pp. 1–6.

48. C. Fang, C. Wan, X. Zhang, S. Okamoto, T. Ma, J. Qin, X. Wang, C. Guo, J. Dong, G. Yu, Z. Wen, N. Tang, S. S. P. Parkin, N. Nagaosa, Y. Lu, X. Han, Observation of the fluctuation spin Hall effect in a low-resistivity antiferromagnet. *Nano Lett.* **23**, 11485–11492 (2023).

49. M. M. H. Polash, D. Vashaee, Anomalous thermoelectric transport properties of Fe-rich magnetic FeTe. *Phys. Status Solidi RRL* **15**, 2100231 (2021).

50. O. J. Amin, A. Dal Din, E. Golias, Y. Niu, A. Zakharov, S. C. Fromage, C. J. B. Fields, S. L. Heywood, R. B. Cousins, F. Maccherozzi, J. Krempaský, J. H. Dil, D. Kriegner, B. Kiraly, R. P. Campion, A. W. Rushforth, K. W. Edmonds, S. S. Dhesi, L. Šmejkal, T. Jungwirth, P. Wadley, Nanoscale imaging and control of altermagnetism in MnTe. *Nature* **636**, 348–353 (2024).

51. Z. Fang, N. Nagaosa, K. S. Takahashi, A. Asamitsu, R. Mathieu, T. Ogasawara, H. Yamada, M. Kawasaki, Y. Tokura, K. Terakura, The anomalous Hall effect and magnetic monopoles in momentum space. *Science* **302**, 92–95 (2003).

52. D. Telesca, Y. Nie, J. I. Budnick, B. O. Wells, B. Sinkovic, Surface valence states and stoichiometry of non-superconducting and superconducting FeTe films. *Surf. Sci.* **606**, 1056–1061 (2012).

53. U. R. Singh, R. Aluru, Y. Liu, C. Lin, P. Wahl, Preparation of magnetic tips for spin-polarized scanning tunneling microscopy on $Fe_{1+y}Te$. *Phys. Rev. B* **91**, 161111(R) (2015).

54. J. Moon, Q. Zou, H. Zhang, O. M. J. van ‘t Erve, N. G. Combs, L. Li, C. H. Li, Magnetic field-induced spin nematic phase up to room temperature in epitaxial antiferromagnetic FeTe thin films grown by molecular beam epitaxy. *ACS Nano* **17**, 16886–16894 (2023).

55. D. Telesca, Y. Nie, J. I. Budnick, B. O. Wells, B. Sinkovic, Impact of valence states on the superconductivity of iron telluride and iron selenide films with incorporated oxygen. *Phys. Rev. B* **85**, 214517 (2012).

56. J. E. Dill, C. F. C. Chang, D. Jena, H. G. Xing, Two-carrier model-fitting of Hall effect in semiconductors with dual-band occupation: A case study in GaN two-dimensional hole gas. *J. Appl. Phys.* **137**, 025702 (2025).
